# Maternal Dietary Patterns During Pregnancy and Child Autism-Related Traits in the Environmental Influences on Child Health Outcomes Consortium

**DOI:** 10.3390/nu16223802

**Published:** 2024-11-06

**Authors:** Rachel Vecchione, Matt Westlake, Megan G. Bragg, Juliette Rando, Deborah H. Bennett, Lisa A. Croen, Anne L. Dunlop, Assiamira Ferrara, Monique M. Hedderson, Jean M. Kerver, Brian K. Lee, Pi-I D. Lin, Irva Hertz-Picciotto, Rebecca J. Schmidt, Rita S. Strakovsky, Kristen Lyall

**Affiliations:** 1 AJ Drexel Autism Institute, Drexel University, Philadelphia, PA 19104, USA; rv422@drexel.edu (R.V.); mb4366@drexel.edu (M.G.B.); jr3639@drexel.edu (J.R.); 2 RTI International, Research Triangle Park, NC 27709, USA; mwestlake@rti.org; 3 Department of Public Health Sciences, School of Medicine, University of California, Davis, CA 95616, USA; dhbennett@ucdavis.edu (D.H.B.); iher@ucdavis.edu (I.H.-P.); rjschmidt@ucdavis.edu (R.J.S.); 4 Division of Research, Kaiser Permanente Northern California, Oakland, CA 94612, USA; lisa.a.croen@kp.org (L.A.C.); assiamira.ferrara@kp.org (A.F.); monique.m.hedderson@kp.org (M.M.H.); 5 Department of Gynecology and Obstetrics, Emory University School of Medicine, Atlanta, GA 30322, USA; amlang@emory.edu; 6 Departments of Epidemiology & Biostatistics and Pediatrics & Human Development, College of Human Medicine, Michigan State University, East Lansing, MI 48824, USA; kerverje@msu.edu; 7 Department of Epidemiology and Biostatistics, Dornsife School of Public Health, Drexel University, Philadelphia, PA 19104, USA; bkl29@drexel.edu; 8 Department of Population Medicine, Harvard Medical School and Harvard Pilgrim Health Care Institute, Boston, MA 02215, USA; p_lin@hphci.harvard.edu; 9 The MIND (Medical Investigations of Neurodevelopmental Disorders) Institute, University of California, Davis, Sacramento, CA 95817, USA; 10 Department of Food Science and Human Nutrition, College of Agriculture & Natural Resources, Michigan State University, East Lansing, MI 48824, USA; strakovs@msu.edu

**Keywords:** dietary patterns, prenatal diet, autism spectrum disorders, Social Responsiveness Scale, HEI, AHEI-P, EDIP

## Abstract

We examined relationships between prenatal dietary patterns and child autism-related outcomes, including parent-reported clinician diagnoses of autism spectrum disorder (ASD) and Social Responsiveness Scale (SRS-2) scores, in up to 6084 participants (with analytic samples ranging from 1671 to 4128 participants) from 14 cohorts in the Environmental Influences on Child Health Outcomes (ECHO) consortium. Associations between quartiles of the Healthy Eating Index (HEI-2015), the Alternative Healthy Eating Index modified for Pregnancy (AHEI-P), and the Empirical Dietary Inflammatory Pattern (EDIP), calculated based on reported prenatal diet, and outcomes were examined using crude and multivariable regression (quantile for SRS scores and logistic for diagnosis). In adjusted models, the higher quartile of prenatal HEI score was associated with lower SRS scores (Q4 vs. Q1 β for median quantile = −3.41 95% CI = −5.15, −1.26). A similar association was observed for the AHEI-P score when adjusting for total calories (Q4 vs. Q1 β = −2.52 95% −4.59, −0.45). There were no significant associations of prenatal diet with ASD diagnosis. Findings from this large U.S.-based study do not suggest strong associations between prenatal dietary patterns and ASD-related outcomes, although subtle associations with broader traits suggest the need to further consider how prenatal diet may relate to ASD-related phenotypes.

## 1. Introduction

Diet during pregnancy plays a major role in fetal development, as evidenced by the effects of overall undernutrition on birth weight, famine on offspring schizophrenia (historical evidence), folic acid deficiency on neural tube defects, and fish intake on neurocognitive outcomes [1,2,3,4]. Prior work has shown inverse associations between several dietary factors and specific neurodevelopmental conditions, including one of the most common neurodevelopmental conditions that is increasing in prevalence: autism spectrum disorder (ASD) [5]. ASD is a neurodevelopmental condition that affects social communication and behaviors and presents in a wide spectrum of behaviors, abilities, and challenges [6]. Evidence suggests that ASD originates prenatally, with both genetic and environmental factors contributing to its complex etiology [7,8]. Prenatal diet may be linked with ASD via several potential mechanisms, including impacts on DNA methylation, inflammation, and oxidative stress, as summarized in several review papers [5,9,10].

Previous work examining relationships between prenatal diet and ASD has largely focused on individual foods, such as fish intake, or individual nutrients, such as folic acid [5]. However, investigating dietary patterns, which summarize regular intake across many foods and nutrients, provides the opportunity to assess overall maternal diet, perhaps allowing for a more complete picture of associations. In summarizing typical dietary behaviors, dietary patterns present the potential to capture more facets of dietary intake than single nutrients alone, including intake across foods and combined effects across nutrients. Dietary patterns also present the potential for increased translation and public health communication of findings around the ways that people typically eat. Several well-established dietary patterns exist, including, among others, the Healthy Eating Index (HEI) and the Alternative Healthy Eating Index (AHEI), which capture adherence to U.S. dietary recommendations, as well as data-derived patterns and those that summarize diets linked to specific health-related outcomes or pathways [11,12,13,14,15,16,17,18,19,20,21,22,23]. Certain dietary patterns, such as the Empirical Dietary Inflammatory Pattern (EDIP), also have been derived to specifically capture mechanisms such as inflammation [22,24,25,26,27], of interest here given the strong evidence for immune aberrations in ASD etiology.

A few studies to date have examined dietary patterns during pregnancy in relation to ASD. Most of these studies have reported inverse associations with diets that may be characterized as “healthy” [28,29,30,31,32]. The largest and most recent of these was conducted in two well-characterized European cohorts and reported a reduction in the odds of an ASD diagnosis and social communication difficulties with greater adherence during pregnancy to a healthy dietary pattern including a higher intake of fruits, vegetables, fish, nuts, and whole grains and lower intake of red and processed meats [28]. Several other smaller studies have similarly reported a reduced risk of ASD and related traits with healthy maternal diets [29,31,32]. Some studies have also reported higher odds of ASD with high consumption of low-quality diets or those higher in pro-inflammatory foods [30,32]. While the existing literature is generally supportive of a link between what may be considered healthier prenatal diets and a reduced likelihood of ASD-related outcomes, certain gaps or limitations in the existing literature base exist. Several prior studies did not examine established dietary patterns, which can present challenges in comparing findings across studies and may limit public health translation [28,30,31]. In addition, while not a limitation in and of itself, most prior studies have not examined multiple ASD-related outcomes, which may present the opportunity to capture subtler effects of the diet or multiple dimensions of ASD [29,30,31]. Finally, most studies to date have relied on small samples with limited diversity [29,30,31].

To address these gaps in our understanding of the relationship between patterns of maternal dietary intake during pregnancy and ASD-related outcomes, we used data from the Environmental influences on Child Health Outcomes (ECHO) consortium, a large and socioeconomically and demographically diverse U.S.-wide sample offering unique opportunities for the study of the prenatal diet and child outcomes [33,34]. Our goals were to examine three established dietary patterns capturing both “healthy” and “unhealthy” aspects of the diet in relation to an ASD diagnosis and quantitative ASD-related traits. Given the prior literature, we hypothesized that adherence to a healthy diet during pregnancy would reduce the likelihood of ASD-related traits and diagnosis, while diets characterized by a higher intake of pro-inflammatory foods would increase the likelihood of ASD and related traits.

## 2. Materials and Methods

### 2.1. Study Population

The study population was drawn from the ECHO program. In its first seven-year cycle, ECHO began as a consortium of 69 existing cohorts from across the U.S. ECHO participants include over 60,000 U.S. children, including members of underserved populations. The ECHO program seeks to better understand perinatal and early childhood environmental exposures and their impact on child health outcomes. ECHO research sites include those with participants recruited from the general population, as well as several study types representing populations with an increased likelihood of ASD [33,35]. These include preterm birth cohorts, as well as cohorts that recruited younger siblings of an existing child with ASD (termed high-familial-likelihood cohorts). To be eligible for the current analyses, participants were required to have, as of 1 March 2023, available prenatal dietary data (to calculate at least one dietary pattern of interest) and data on ASD outcomes (in the form of either a Social Responsiveness Scale (SRS-2) score, further described below, or information on ASD diagnosis). In addition, only singleton pregnancies were included given the potential for differences in prenatal diet and a higher likelihood of ASD with multiple and higher-order births. If multiple children from the same mother participated in ECHO, one child was selected at random for inclusion in this study. Figure S1 shows the selection criteria for these analyses. All participants provided informed consent.

### 2.2. Dietary Assessment

Dietary information was collected from individual cohorts according to existing protocols prior to ECHO participation (see Table S1 for a list of dietary measures used by each cohort). Cohort dietary data included here were based primarily on three different food frequency questionnaires (FFQs), the Block FFQ, the Harvard FFQ, and the Diet History Questionnaire II (DHQ2), with all but one included study (ReCHARGE) assessing diet prospectively during pregnancy. For one dietary pattern, data were also used from the Automated Self-Administered 24 h dietary assessment tool (ASA-24). All three FFQs asked about the regular intake of over 100 food items over a period of time (since becoming pregnant), while the ASA-24 derived nutrient information based on the reported intake of all foods over a 24 h period, averaged across several reports. The Block FFQ provides nine options for food intake frequency and three for portion size, which were selected based on data reported in NHANES II [36]. The Harvard FFQ is a semi-quantitative FFQ that asks participants to report their usual intake of foods with nine options for frequency of intake (and portion size is included as part of the food item itself) [37]. The Diet History Questionnaire II (DHQ2) is a modified version of the National Cancer Institute’s Dietary History Questionnaire that asks participants to report their usual intake with nine options for frequency of intake and three options for portion size at each time of consumption [38]. If multiple prenatal timepoints of dietary assessment were available for an individual, the one collected earliest in pregnancy was chosen due to higher data availability, prior evidence supporting a high similarity of the diet across pregnancy, and greater evidence for associations of the early pregnancy diet with ASD-related outcomes [5]. All measures used represent validated dietary assessments [34,39] with evidence for their comparability in estimating intakes of key nutrients and overall dietary patterns [32,34,39].

### 2.3. Dietary Pattern Indices

Dietary patterns included in the analyses here were selected as outlined in the Introduction on the basis of prior work, and based on the feasibility of assessment in ECHO with the existing data. Dietary patterns (below) were calculated based on reported intakes from the dietary assessments described above. Given the differences in dietary data needed to calculate the dietary pattern scores, not all cohorts contributed to all dietary pattern analyses, and each dietary pattern was analyzed in association with ASD-related outcomes separately (see Table S1). Specifically, the Empirical Dietary Inflammatory Pattern (EDIP) was derived from cohorts with Block and DHQ FFQ data only, and the Alternative Healthy Eating Index (AHEI) modified for pregnancy (AHEI-P) was derived from cohorts with Harvard and DHQ FFQ data only, while the Healthy Eating Index (HEI) was derived from Block, Harvard, and DHQ FFQ data, as well as 24 h dietary recall data (ASA24), each following standard methods [34].

The EDIP characterizes the overall inflammatory potential of the diet and was derived using reduced rank regression to identify food groups most predictive of markers of inflammation (specifically, interleukin-6 (IL-6), C-reactive protein (CRP), and tumor necrosis factor α receptor 2 (TNFαR2)). A cumulative score is derived based on the intake of 18 identified food groups, with a higher score representing a more pro-inflammatory (“unhealthy”) diet [22,23]. In contrast, the HEI and AHEI-P are dietary patterns that characterize adherence to established dietary guidelines, with higher scores representing better adherence to guidelines (or generally representative of a “healthier” diet) [12,13]. Here, we used the version of the HEI based on the 2015 Dietary Guidelines for Americans. The HEI-2015 includes 13 components (total fruits, whole fruits, total vegetables, greens and beans, whole grains, dairy, total protein foods, seafood and plant proteins, fatty acids, refined grains, sodium, added sugars, and saturated fats). HEI scores range from 0 to 100. Some components are coded from 0 to 5 and some from 0 to 10. Additionally, some are coded directly (higher score meaning higher intake) and some are reverse coded (higher score meaning lower intake) [11]. The AHEI is a modified version of the HEI that was developed to better predict chronic disease risk [13,14]. The AHEI-2010 is scored out of 110 and includes 11 components: vegetables, fruit, whole grains, sugar-sweetened beverages and fruit juice, nuts, legumes, and vegetable protein, red/processed meats, trans fats, long-chain (n-3) fats, PUFAs, sodium, and moderate alcohol intake [14]. All components are scored from 0 to 10, and similar to the HEI, some components are reverse coded. The AHEI-P is a version of the AHEI modified with pregnancy-specific recommendations and is scored from 0 to 90. The AHEI-P includes 9 components: vegetables, fruit, ratio of white to red meat, fiber, trans fats, ratio of polyunsaturated to saturated fat, calcium, folate, and iron [15].

### 2.4. Outcome Assessment

We examined two related outcomes in the child: quantitative, broader ASD-related trait scores, as captured by the Social Responsiveness Scale (SRS-2), and ASD diagnosis according to a parent report of a clinician diagnosis (with some diagnoses confirmed with medical record abstraction or clinical assessment depending on the cohort). Not all participants had both SRS scores and an ASD diagnosis; thus, the sample size varied across analyses. The Social Responsiveness Scale (SRS-2) is a 65-item validated measure of ASD-related traits that yields a single quantitative score [40,41,42]. Higher SRS scores indicate a greater degree of ASD-related traits. Primary analyses used total raw SRS scores from parent reports (primary respondent: mother). Raw scores were used for primary analyses to facilitate a comparison with previous work and given the use of multiple SRS form types here. Raw scores range from 0–195 and can also be converted into normed T-scores to facilitate clinical guidance. The SRS has a preschool (age 2.5–4.5 years) and school age (age 4–<18 years) form for child assessment; these forms differ only on a handful of items in the use of age-appropriate examples and have been shown to be correlated in ECHO [40,41,42,43,44]. In addition, ECHO includes the collection of a shortened 16-item form as an option; this short form includes 16 of the original 65 SRS items and can be normed to total scores that have been previously validated and shown to have the same distribution properties, predictive abilities, and associations with risk factors as full 65-item scores [45,46,47]. Forms with >15% of items missing or an age of administration that was <2.5 or >18 were excluded.

### 2.5. Statistical Analysis

Descriptive statistics were calculated to summarize participant characteristics and compare dietary pattern scores across cohorts and outcomes. For the examination of associations with outcomes, dietary patterns were parameterized in quartiles, with the lowest quartile as the reference. Associations between quartiles of each dietary pattern and SRS scores were examined using crude and multivariable quantile regression. Quantile regression is similar to linear regression, but rather than modeling associations as the mean of the outcome as in linear regression, it enables exploration across different quantiles of the outcome. Rather than linear regression, quantile regression provides the ability to better account for different distributions at specific quantiles and is more robust to data that are not normally distributed [48]. Here, the primary analyses were fixed at the 50th percentile of SRS total raw score, though associations modeled at other deciles were examined in secondary analyses. Associations between quartiles of each dietary pattern score and ASD diagnosis were examined using crude and multivariable logistic regression.

Adjustment for potential confounding factors, including sociodemographic factors or other ASD risk factors that may also relate to diet, is an important component of the study of prenatal dietary patterns and child outcomes. Here, potential covariates were selected on the basis of having existing evidence for associations with diet and/or ASD. These covariates in all final adjusted models included maternal age (continuous) [49] and maternal pre-pregnancy BMI (continuous) [50] (given evidence for positive associations with each of these and ASD, and potential for differences in diet by age and BMI); maternal sociodemographic factors (given evidence for differences in diet and ASD by these) [51], including ethnicity/race (non-Hispanic white, non-Hispanic Black, Hispanic, other) and maternal education (less than high school, high school/GED, some college/associates degree/trade school, bachelor’s degree, graduate degree); maternal smoking (yes, no) [52] (given evidence for links with both ASD and diet); as well as child sex (male, female) and child year of birth (1998–2004, 2005–2009, 2010–2014, 2015+) given the strong sex ratio in ASD and time trends in ASD prevalence [53]. We also included adjustment for cohort type (high-familial-ASD-probability cohort/general population), given differences in the likelihood of ASD outcomes in these populations. For AHEI-P and EDIP, we also tested energy adjustment (not included in HEI models because this pattern is derived incorporating food intake per 1000 calories). We additionally included maternal race/ethnicity, recognizing it as a social construct associated with experiences of structural inequities, racism, and environmental injustice. Race/ethnicity has been linked to both differences in maternal diet and disparities in SRS sensitivity and ASD diagnosis. Missingness among covariates was very low (<25%), and multiple imputation using PROC MI with 25 iterations was used to assign values on any missing covariates in the final adjusted models.

In secondary analyses, we examined associations of dietary patterns and quantiles of SRS scores other than the 50th percentile, to assess whether associations may differ at the tails of the outcome distribution (10th, 20th, 70th, and 90th percentiles). Sensitivity analyses included additional adjustment for each of the following separately: dietary assessment method (FFQ type), supplement use during pregnancy (ever), supplement use in the first month of pregnancy, dietary folic acid intake, parity, breastfeeding, income, preterm birth, birth size, and gestational diabetes (GDM), to test the impact of potential residual confounding. These covariates had higher levels of missingness than primary covariates, and alternate missing data strategies were used based on the percent of missing data in order to balance feasibility and reduce potential bias: multiple imputation (for variables with <25% missingness), missingness indicators (25–50% missingness), and subset analyses for variables with higher proportions of missing data (50% to 90% missingness). In additional analyses, we excluded dietary data collected using 24 h recalls and trait scores collected using the preschool age or the 16-item SRS form types. In addition, to assess whether results were driven by a given cohort, we conducted sensitivity analyses excluding individual cohorts one at a time. Finally, in order to facilitate clinical interpretations, we conducted analyses using SRS T-scores rather than SRS raw scores.

## 3. Results

Primary analyses included up to 6084 mother–child dyads from across 14 cohorts, with sample size differing across the models for a given dietary pattern and ASD-related outcome, as shown in Figure S1. Participant characteristics are shown in Table 1. Of the eligible study participants, 95% were from cohorts drawn from the general population. Across the included cohorts, most maternal participants were white, non-Hispanic, younger than 34 years (at time of birth of the child), had at least a bachelor’s degree, and did not smoke during pregnancy. Of child participants, 7% had an ASD diagnosis (with a higher-than-general-population prevalence owing to the inclusion of a case–control and several high-familial-ASD-probability cohorts). The mean total raw SRS score was 29.3 (SD = 22.5).

Demographic characteristics of participants were broadly comparable across dietary patterns for both outcome datasets (those participants with SRS outcome data and those participants with ASD outcome data) (Tables S2 and S3). However, across outcome analytic samples (those participants with each dietary pattern exposure within each outcome dataset), modest differences in participant characteristics across exposure datasets were evident; those with data available on AHEI-P scores were more likely to be white, non-Hispanic, and have a BMI in the normal range, and were less likely to be <28 years old, as compared to participants with data on HEI and EDIP scores. These modest differences may be because data for AHEI-P analyses were drawn from two cohorts from the northeast, whereas other cohorts were more geographically diverse. The average maternal caloric intake was comparable across outcome analytic samples. In addition, dietary pattern scores were comparable across outcome analytic samples (Table S4).

In adjusted quantile regression analyses of associations between maternal prenatal dietary patterns and child SRS raw scores, higher HEI scores (quartiles 2, 3, and 4) were associated with lower SRS raw scores (for example, HEI Q4 vs. Q1 β for median quantile = −3.41 95% CL = −5.15, −1.26; Table 2). Somewhat more modest inverse associations were also observed with higher quartiles of AHEI-P scores, which were statistically significant when including adjustment for total calories (Q4 vs. Q1 β = −2.52 95% −4.59, −0.45). EDIP was not associated with child SRS raw scores in adjusted models. In secondary analyses examining results across different quantiles of SRS scores, the results were similar, and there was no evidence that associations were stronger at higher quantiles (Table S5).

When examining associations with ASD diagnosis (Table 3), we did not observe evidence for significant associations of HEI or AHEI-P with ASD diagnosis. The estimate for the highest quartile of the EDIP dietary pattern was modestly increased, but confidence intervals were fairly wide and included the null.

Associations were robust to several sensitivity analyses, including adjustment for other covariates (Tables S6 and S7), leaving out individual cohorts one at a time (Figure S2), and exclusion of different SRS form types (Table S8), with the exception of the HEI and SRS association. This association was attenuated to the null when adjusting for diet form type, excluding 24 h recall data, or excluding SRS preschool and short forms (of which 38% were preschool forms and 62% were the short forms). However, each of these analyses was tied to the cohort, with two of the largest cohorts including either 24 h recall data or SRS short-form data. Removal of these two cohorts (CANDLE n = 638, Healthy Start n = 434) attenuated this association (Figure S2). Findings were consistent for primary analyses when using SRS T-scores instead of SRS raw scores (Table S9).

## 4. Discussion

Overall, in this large, diverse U.S. sample of pregnant individuals and their children, we did not see strong evidence for associations of the prenatal dietary pattern score with ASD diagnosis, though we did find some evidence that better adherence to healthier diets was associated with modest decreases in ASD-related traits. Somewhat mixed findings across dietary patterns and ASD outcomes suggest the need for continued study, perhaps considering additional dietary patterns or utilizing other approaches to consider combined effects of prenatal intake of different foods and nutrients on child neurodevelopmental outcomes.

Although few studies have examined the relations of prenatal dietary patterns with child neurodevelopmental outcomes, our findings are broadly consistent with the prior literature. While this is the first study to suggest an inverse association between the HEI dietary pattern and SRS scores, several studies have reported inverse associations of maternal ‘healthy’ or ‘balanced’ dietary patterns with child ASD-related traits. Most notably, the largest study to date on the topic, including 84,548 participants (942 with autism) from the Norwegian Mother, Father, and Child Cohort Study (MoBa) and 11,670 participants (544 with social communication difficulties) from the Avon Longitudinal Study of Parents and Children (ALSPAC), found that high adherence during pregnancy to a data-derived healthy dietary pattern was associated with reduced odds of ASD diagnosis and social communication difficulties [28]. Specifically, one cohort study in the U.S. (including 325 participants drawn from the Newborn Epigenetics Study (NEST)) reported an inverse association between maternal adherence to the Mediterranean diet (which is characterized by a low meat intake and higher intake of fruits, vegetables, and fatty acids) during pregnancy and ASD-related traits as captured by a composite score derived from questions from the Infant Toddler Social and Emotional Assessment [29]. In addition, two separate small case–control studies in China have reported an increase in odds of ASD with an unbalanced/unhealthy prenatal diet [30], and a reduction in the odds of ASD with a high consumption of fruit and fish during pregnancy [31].

While our findings of an inverse association between HEI and ASD-related traits are broadly supported by some prior evidence, several considerations are worth noting. First, associations were attenuated to null in several sensitivity analyses, which may have been a result of the reduced sample size and power. Removal of two of the larger cohorts (Conditions Affecting Neurocognitive Development and Learning in Early Childhood (CANDLE; n = 638) and HEALTHY START (n = 434)) attenuated the association in leave-one-out analyses. Additionally, we adjusted for the dietary intake form type. The further adjustment for diet form type primarily reflects the removal of a large cohort (HEALTHY START) that used 24 h recalls to collect its dietary data. Furthermore, associations were attenuated when SRS preschool and short forms were excluded, with most of these exclusions resulting from use of the short form (which was used for CANDLE study participants). The preschool and short versions of the SRS have been validated and have been shown to be comparable with the full score [43]. The results of sensitivity analyses suggest the association may not be robust to all populations or settings, that this association was biased by the inclusion of 24 h recall data, or that the reduced sample size impacted our ability to detect the association.

Second, while both the HEI and AHEI-P capture the adherence to a healthy diet, and the findings were similar across these, our results were slightly stronger for the HEI than the AHEI-P. Prior results from a study conducted by members of our team using data from two U.S. cohorts (including 154 individuals from the ECHO Early Autism Risk Longitudinal Investigation (EARLI) cohort, as well as 727 participants from the Nurses’ Health Study II (NHSII), which is not included in ECHO) also did not find strong associations with the AHEI-P, although they did not examine the HEI [32]. Given that the AHEI-P includes specific modifications for pregnancy, such as the inclusion of folic acid, it is possible that HEI associations were confounded by folic acid intake or other components of the AHEI-P not included in the HEI, such as fiber or iron. When we adjusted HEI models for folic acid intake or prenatal supplement use, given prior evidence for reductions in the risk of ASD with folic acid supplementation [5], the results for the association between HEI and SRS were materially unchanged, but information on supplement use was not well captured in ECHO. A related point is that minor differences in food group composition across patterns may have contributed to differences. In addition, although we adjusted for the cohort in all analyses, fewer cohorts contributed to the AHEI-P pattern than to the HEI pattern, and those included in the AHEI-P analyses were also less demographically and geographically diverse than the cohorts that contributed to the other dietary patterns.

An additional consideration is that we did not observe consistent patterns of association across SRS scores and ASD diagnosis. Higher HEI scores (and to a lesser extent AHEI-P scores) were associated with lower SRS scores but were not associated with reduced odds of ASD diagnosis. The differences in findings across these outcomes could point to a potential role of healthy prenatal dietary patterns having minor influences on subclinical or broader traits, rather than diagnosis itself. Alternatively, while parent-reported diagnosis of ASD was validated in several cohorts and the validity of the SRS has been supported, we cannot rule out measurement error in outcome classifications contributing to discrepancies across diet associations with the outcome. We did not observe significant differences in outcomes in the cohorts contributing to the analyses, suggesting this was not a large driver of our findings. Continued research comparing outcome metrics, and considering relationships with additional neurodevelopmental constructs, is needed to better understand the specificity of potential associations.

In addition to some evidence for associations between healthy diets and ASD-related traits, we also observed some evidence for a modest—but not statistically significant—increase in odds of ASD with a more pro-inflammatory diet as measured by the EDIP. This finding is broadly consistent with a study conducted by members of our team including 154 participants from EARLI and 727 participants from NHSII. Our prior study observed a non-significant but positive association between the Western dietary pattern, which is characterized by higher meat and processed food intake, and ASD-related traits as measured by SRS [32]. Of note, while there was some minor overlap in participants across these studies (EARLI was included in both analyses), this overlap consisted of a small number of participants, and removal of EARLI from analyses here did not significantly influence our findings.

Several mechanisms could underlie associations between these dietary patterns and ASD outcomes. Given prior evidence for the role of prenatal maternal immune activation in pregnancy, we hypothesized that inflammation may be a key mechanism [54,55,56]. Adherence to the HEI has been associated with decreased inflammatory markers [57], and thus HEI scores may relate to the neurodevelopmental outcome via an anti-inflammatory pathway, though we did not see strong evidence of associations with the EDIP, which captures a pro-inflammatory diet. The EDIP score was not created in a pregnant population, and inflammatory markers are known to differ during pregnancy. For example, IL-6 levels have been found to differ between pregnant and non-pregnant women and between women at different points during pregnancy [58]. Other, more direct mechanisms related to fetal neurodevelopment could link a healthy dietary intake to outcomes. For example, prenatal adherence to the HEI has been found to be positively associated with the development of white matter in fetal brains [59], and differential development of white matter has been associated with ASD [60].

Our study has several strengths. We included a large demographically and geographically diverse study population, and we were able to assess associations with two ASD-related outcomes. We also examined established, clearly defined dietary patterns, which facilitate a comparison across studies and clear communication of public health recommendations that may help to inform dietary guidelines. However, there were also several limitations to this study to consider in interpreting the results and guiding future work. First, different dietary assessment measures were used in different cohorts, and different cohorts were included in analyses of different patterns and outcomes. While validated measures were used, and participants were ranked, the self-reported diet is known to be measured with error. Although cohort characteristics and exposures were broadly comparable across analytic samples, it is possible that individual cohort characteristics or measurement error may have differentially contributed to different analyses. Second, we were not able to examine the diet in more specific time windows than pregnancy overall, which means that associations restricted to a narrower critical window may have been missed. Third, we cannot rule out the role of potential residual confounding. Although we tested adjustment of several additional factors in models, including prenatal supplement use, and sensitivity analyses did not meaningfully change the results, few cohorts had data available on the timing and initiation of prenatal supplement use. Furthermore, we cannot rule out unmeasured confounding by other health-promoting behaviors that were not captured here, which may be tied to greater adherence to dietary guidelines. Fourth, another potential limitation is that ASD diagnosis was parent-reported. However, in some cohorts, diagnosis was confirmed by clinical diagnosis/medical record abstractions. Fifth, while ECHO represents a more diverse population than many prior studies, it does not necessarily represent the U.S. general population as a whole, and the results here may not be generalized to other populations. Finally, while not the focus of this work, certain foods also represent sources of exposure to chemical contamination, such as via food preparation and storage, and how this may impact associations was not examined here. Future research should seek to address these limitations and clarify the role of potential unmeasured factors, timing, and joint effects in the study of prenatal diet and ASD.

## 5. Conclusions

In this large U.S. consortium study, we found some evidence that better adherence to U.S. dietary guidelines during pregnancy is associated with modest reductions in traits related to ASD; however, this finding was not robust to all analyses and requires further study. We did not see strong evidence for associations of the outcomes under study with unhealthy or pro-inflammatory diets. Additional investigation is needed to better understand how the prenatal diet overall, and specific aspects of it, influences outcomes across neurodevelopmental constructs.

## Figures and Tables

**Table 1:** Basic characteristics of the study populations from 14 ECHO cohorts (*n* = 6084).

Characteristics	Participants with any Data Available for ASD Diagnosis and/or SRS Score
	N (%)
Cohort Type	
High Familial Likelihood	309(5%)
Population-based	5775(95%)
Maternal and Child Characteristics	
Maternal Race	
Asian or Pacific Islander	491(8%)
Black/African American	1222(20%)
Native American or Native Alaskan	31(1%)
White	3728(61%)
Multiple/Other Race	374(6%)
Unknown/Missing	238(4%)
Maternal Ethnicity	
Hispanic/Latino	1029(17%)
Not Hispanic/Latino	5037(83%)
Missing	18(0%)
Maternal Age, Years	
<18–28 Years	1981(33%)
29–34 Years	2551(42%)
35–40 Years	1371(23%)
41+ years	181(3%)
Maternal Education	
Less than High School	309(5%)
HS Degree, GED, or Equivalent	1011(17%)
Some College, No Degree, Assoc/Trade	1410(23%)
Bachelor’s Degree (BA, BS)	1783(29%)
Masters, Prof, or Doctorate Degree	1491(25%)
Missing	80(1%)
Pre-Pregnancy BMI, kg/m^2^	
<18.5	173(3%)
18.5–24.9	2837(47%)
25–29.9	1544(25%)
≥30	1466(24%)
Missing	64(1%)
Prenatal Smoking	
Active	322(5%)
Not Active	5730(94%)
Missing	32(1%)
Ever Breastfeed	
Yes	4259(70%)
No	117(2%)
Missing	1708(28%)
Prenatal Vitamin Use	
Yes	2947(48%)
No	178(3%)
Missing	2959(49%)
Prenatal Vitamin Use (First Month)	
Yes	296(5%)
No	190(3%)
Missing	5598(92%)
Child Sex	
Male	3195(53%)
Female	2889(47%)
Child Year of Birth	
1999–2004	671(11%)
2005–2009	805(13%)
2010–2014	2674(44%)
2015+	1934(32%)
Birthweight	
Small for Gestational Age	326(5%)
Normal for Gestational Age	4573(75%)
Large for Gestational Age	996(16%)
Missing	189(3%)
ASD Diagnosis	
Yes	441(7%)
No	5381(88%)
Missing	262(4%)
Total SRS Raw Score	
SRS Not Available	1537(25%)
SRS Available	4547(75%)
	Mean (Std)
Parity (Prior to Current Pregnancy)	0.83(0.98)
Total Caloric Intake, kcal	2065(1053)
SRS Score	29.3(22.5)

**Table 2:** Association between maternal dietary patterns during pregnancy and child SRS raw scores.

	n	Crude (ß, 95% CI)	Adjusted (ß, 95% CI)	Test of Trend (p)	Energy Adjusted (ß, 95% CI)	Test of Trend (p)
EDIP	2433					
Q1	608	0 (reference)	0 (reference)	0.44	0 (reference)	0.20
Q2	608	−1.00 (−14.10, 17.10)	−1.25 (−3.73, 0.65)		−1.20 (−3.79, 0.54)	
Q3	609	−2.00 (−14.60, 15.10)	−0.42 (−3.44, 2.11)		−0.40 (−3.43, 2.16)	
Q4	608	−4.00 (−17.10, 13.10)	0.64 (−1.91, 2.85)		0.72 (−2.76, 2.67)	
AHEIP	1671					
Q1	417	0 (reference)	0 (reference)	0.02	0 (reference)	0.02
Q2	418	−2.00 (−15.67, 4.67)	0.00 (−2.56, 1.79)		−0.46 (−2.61, 1.69)	
Q3	418	−4.00 (−20.18, 4.18)	−1.50 (−3.95, 0.86)		−2.12 (−4.73, 0.50)	
Q4	418	−4.00 (−19.18, 3.18)	−1.50 (−3.27, 0.39)		−2.52 (−4.59, −0.45)	
HEI ^1^	2876					
Q1	719	0 (reference)	0 (reference)	0.12	0 (reference)	-
Q2	719	0.00 (−17.09, 13.09)	−1.84 (−3.99, −0.20)		-	
Q3	719	0.00 (−16.09, 15.09)	−4.24 (−6.17, −2.26)		-	
Q4	719	1.00 (−14.09, 14.09)	−3.41 (−5.15, −1.26)		-	

Modeled using quantile regression fixed at 50th percentile of SRS total raw score. Adjusted: maternal age, maternal pre-pregnancy BMI, child sex (male, female), cohort type (familial/not), maternal ethnicity/race (non-Hispanic white, non-Hispanic Black, Hispanic, other), maternal education (less than high school, high school/GED, some college/associates degree/trade school, bachelor’s degree, graduate degree), maternal smoking (yes, no), and child year of birth (1998–2004, 2005–2009, 2010–2014, 2015+). Energy Adjusted: maternal age, maternal pre-pregnancy BMI, child sex (male, female), cohort type (familial/not), maternal ethnicity/race (non-Hispanic white, non-Hispanic Black, Hispanic, other), maternal education (less than high school, high school/GED, some college/associates degree/trade school, bachelor’s degree, graduate degree), maternal smoking (yes, no), child year of birth (1998–2004, 2005–2009, 2010–2014, 2015+), and total caloric intake (kcal). ^1^ HEI food groups are energy scaled and thus adjustment for energy intake was not conducted for HEI.

**Table 3:** Association between maternal dietary patterns and child autism spectrum disorder diagnosis.

	n Case/Total	Crude (OR, 95% CI)	Adjusted (OR, 95% CI)	Test of Trend (p)	Energy Adjusted (OR, 95% CI)	Test of Trend (p)
EDIP	3614					
Q1	90/904	1 (reference)	1 (reference)	0.25	1 (reference)	0.25
Q2	90/901	1.00 (0.74, 1.37)	1.27 (0.88, 1.82)		1.27 (0.88, 1.83)	
Q3	108/906	1.22 (0.91, 1.65)	1.27 (0.89, 1.81)		1.26 (0.88, 1.79)	
Q4	103/903	1.16 (0.86, 1.57)	1.29 (0.89, 1.86)		1.25 (0.83, 1.89)	
AHEIP	1694					
Q1	17/423	1 (reference)	1 (reference)	0.89	1 (reference)	0.89
Q2	16/424	0.94 (0.47, 1.88)	1.14 (0.49, 2.65)		1.19 (0.50, 2.83)	
Q3	15/424	0.88 (0.43, 1.78)	1.23 (0.52, 2.90)		1.20 (0.50, 2.90)	
Q4	10/423	0.58 (0.26, 1.28)	0.89 (0.35, 2.28)		0.85 (0.31, 2.32)	
HEI ^1^	4128					
Q1	65/1032	1 (reference)	1 (reference)	0.81	1 (reference)	-
Q2	116/1032	1.88 (1.37, 2.59)	1.04 (0.71, 1.51)		-	
Q3	109/1032	1.76 (1.28, 2.42)	0.92 (0.62, 1.35)		-	
Q4	93/1032	1.47 (1.06, 2.05)	0.99 (0.66, 1.49)		-	

Models using logistic regression. Adjusted: maternal age, maternal pre-pregnancy BMI, child sex (male, female), cohort type (familial/not), maternal ethnicity/race (non-Hispanic white, non-Hispanic Black, Hispanic, other), maternal education (less than high school, high school/GED, some college/associates degree/trade school, bachelor’s degree, graduate degree), maternal smoking (yes, no), and child year of birth (1998–2004, 2005–2009, 2010–2014, 2015+). Adjusted: maternal age, maternal pre-pregnancy BMI, child sex (male, female), cohort type (familial/not), maternal ethnicity/race (non-Hispanic white, non-Hispanic Black, Hispanic, other), maternal education (less than high school, high school/GED, some college/associates degree/trade school, bachelor’s degree, graduate degree), maternal smoking (yes, no), child year of birth (1998–2004, 2005–2009, 2010–2014, 2015+), and total caloric intake (kcal). ^1^ HEI food groups are energy scaled and thus adjustment for energy intake was not conducted for HEI.

## Data Availability

Select de-identified data from the ECHO Program are available through NICHD’s Data and Specimen Hub (DASH). Information on study data not available on DASH, such as some indigenous datasets, can be found on the ECHO study DASH webpage.

## Supplementary Materials

The following supporting information can be downloaded at https://www.mdpi.com/article/10.3390/nu16223802/s1, Figure S1: Study participant flowchart across the cohorts; Figure S2: Associations between dietary patterns and SRS scores removing one cohort at a time for (a) SRS:HEI, (b) SRS:AHEIP, (c) SRS:EDIP; (d) ASD:HEI, (e)ASD:AHEIP, (f) ASD:EDIP; Table S1: Diet data source by cohort; Table S2: Participant characteristics by dietary pattern analytic sample among those with SRS outcome data (SRS-Any n = 4547, SRS-HEI n = 2876, SRSAHEI-P n = 1671, SRS-EDIP n = 2433); Table S3: Participant characteristics by dietary pattern analytic sample among those with ASD outcome data (ASD-Any n = 5822, ASD-HEI n = 4128, ASD-AHEI-P n = 1694, ASD-EDIP n = 3614); Table S4: Distribution (mean, SD) of dietary pattern scores across the cohorts included in analytic samples; Table S5: Association between maternal dietary patterns during pregnancy and child SRS raw scores modeled using quantile regression fixed at the 10th, 20th, 70th, and 90th percentiles; Table S6: Association between maternal dietary patterns during pregnancy and child SRS raw scores + adjustment for additional factors; Table S7: Association between maternal dietary patterns during pregnancy and child ASD diagnosis + adjustment for additional factors; Table S8: Association between maternal dietary patterns during pregnancy and child SRS raw scores, excluding SRS preschool and short forms; Table S9: Association between maternal dietary patterns during pregnancy and SRS T-scores.

## References

[1] Susser E.S., Lin S.P. (1992). Schizophrenia after prenatal exposure to the Dutch Hunger Winter of 1944–1945. Arch. Gen. Psychiatry.

[2] De-Regil L.M., Fernández-Gaxiola A.C., Dowswell T., Peña-Rosas J.P. (2010). Effects and safety of periconceptional folate supplementation for preventing birth defects. Cochrane Database Syst. Rev..

[3] Dean S.V., Lassi Z.S., Imam A.M., Bhutta Z.A. (2014). Preconception care: Nutritional risks and interventions. Reprod. Health.

[4] Hibbeln J.R., Spiller P., Brenna J.T., Golding J., Holub B.J., Harris W.S., Kris-Etherton P., Lands B., Connor S.L., Myers G. (2019). Relationships between seafood consumption during pregnancy and childhood and neurocognitive development: Two systematic reviews. Prostaglandins Leukot. Essent. Fatty Acids.

[5] Zhong C., Tessing J., Lee B.K., Lyall K. (2020). Maternal Dietary Factors and the Risk of Autism Spectrum Disorders: A Systematic Review of Existing Evidence. Autism Res..

[6] APA (2013). Diagnostic and Statistical Manual of Mental Disorders: DSM V.

[7] Rodier P.M. (2000). The early origins of autism. Sci. Am..

[8] Newschaffer C.J., Croen L.A., Daniels J., Giarelli E., Grether J.K., Levy S.E., Mandell D.S., Miller L.A., Pinto-Martin J., Reaven J. (2007). The epidemiology of autism spectrum disorders. Annu. Rev. Public Health.

[9] DeVilbiss E.A., Gardner R.M., Newschaffer C.J., Lee B.K. (2015). Maternal folate status as a risk factor for autism spectrum disorders: A review of existing evidence. Br. J. Nutr..

[10] Maitin-Shepard M., O’Tierney-Ginn P., Kraneveld A.D., Lyall K., Fallin D., Arora M., Fasano A., Mueller N.T., Wang X., Caulfield L.E. (2024). Food, nutrition, and autism: From soil to fork. Am. J. Clin. Nutr..

[11] Krebs-Smith S.M., Pannucci T.E., Subar A.F., Kirkpatrick S.I., Lerman J.L., Tooze J.A., Wilson M.M., Reedy J. (2018). Update of the Healthy Eating Index: HEI-2015. J. Acad. Nutr. Diet..

[12] Reedy J., Lerman J.L., Krebs-Smith S.M., Kirkpatrick S.I., Pannucci T.E., Wilson M.M., Subar A.F., Kahle L.L., Tooze J.A. (2018). Evaluation of the Healthy Eating Index-2015. J. Acad. Nutr. Diet..

[13] McCullough M.L., Feskanich D., Stampfer M.J., Giovannucci E.L., Rimm E.B., Hu F.B., Spiegelman D., Hunter D.J., Colditz G.A., Willett W.C. (2002). Diet quality and major chronic disease risk in men and women: Moving toward improved dietary guidance. Am. J. Clin. Nutr..

[14] Chiuve S.E., Fung T.T., Rimm E.B., Hu F.B., McCullough M.L., Wang M., Stampfer M.J., Willett W.C. (2012). Alternative dietary indices both strongly predict risk of chronic disease. J. Nutr..

[15] Rifas-Shiman S.L., Rich-Edwards J.W., Kleinman K.P., Oken E., Gillman M.W. (2009). Dietary quality during pregnancy varies by maternal characteristics in Project Viva: A US cohort. J. Am. Diet. Assoc..

[16] Hu F.B., Rimm E., Smith-Warner S.A., Feskanich D., Stampfer M.J., Ascherio A., Sampson L., Willett W.C. (1999). Reproducibility and validity of dietary patterns assessed with a food-frequency questionnaire. Am. J. Clin. Nutr..

[17] Lopez-Garcia E., Schulze M.B., Fung T.T., Meigs J.B., Rifai N., Manson J.E., Hu F.B. (2004). Major dietary patterns are related to plasma concentrations of markers of inflammation and endothelial dysfunction. Am. J. Clin. Nutr..

[18] Filippou C.D., Tsioufis C.P., Thomopoulos C.G., Mihas C.C., Dimitriadis K.S., Sotiropoulou L.I., Chrysochoou C.A., Nihoyannopoulos P.I., Tousoulis D.M. (2020). Dietary Approaches to Stop Hypertension (DASH) Diet and Blood Pressure Reduction in Adults with and without Hypertension: A Systematic Review and Meta-Analysis of Randomized Controlled Trials. Adv. Nutr..

[19] National Heart, Lung, and Blood Institute (NHLBI) (2021). DASH Eating Plan.

[20] Vogt T.M., Appel L.J., Obarzanek E., Moore T.J., Vollmer W.M., Svetkey L.P., Sacks F.M., Bray G.A., Cutler J.A., Windhauser M.M. (1999). Dietary Approaches to Stop Hypertension: Rationale, design, and methods. DASH Collaborative Research Group. J. Am. Diet. Assoc..

[21] Shivappa N., Steck S.E., Hurley T.G., Hussey J.R., Hébert J.R. (2014). Designing and developing a literature-derived, population-based dietary inflammatory index. Public Health Nutr..

[22] Tabung F.K., Smith-Warner S.A., Chavarro J.E., Fung T.T., Hu F.B., Willett W.C., Giovannucci E.L. (2017). An Empirical Dietary Inflammatory Pattern Score Enhances Prediction of Circulating Inflammatory Biomarkers in Adults. J. Nutr..

[23] Tabung F.K., Smith-Warner S.A., Chavarro J.E., Wu K., Fuchs C.S., Hu F.B., Chan A.T., Willett W.C., Giovannucci E.L. (2016). Development and Validation of an Empirical Dietary Inflammatory Index. J. Nutr..

[24] Nardone S., Elliott E. (2016). The Interaction between the Immune System and Epigenetics in the Etiology of Autism Spectrum Disorders. Front. Neurosci..

[25] Han V.X., Patel S., Jones H.F., Dale R.C. (2021). Maternal immune activation and neuroinflammation in human neurodevelopmental disorders. Nat. Rev. Neurol..

[26] Beversdorf D.Q., Stevens H.E., Jones K.L. (2018). Prenatal Stress, Maternal Immune Dysregulation, and Their Association with Autism Spectrum Disorders. Curr. Psychiatry Rep..

[27] Gonzalez-Nahm S., Mendez M., Robinson W., Murphy S.K., Hoyo C., Hogan V., Rowley D. (2017). Low maternal adherence to a Mediterranean diet is associated with increase in methylation at the MEG3-IG differentially methylated region in female infants. Environ. Epigenet..

[28] Friel C., Leyland A.H., Anderson J.J., Havdahl A., Brantsæter A.L., Dundas R. (2024). Healthy Prenatal Dietary Pattern and Offspring Autism. JAMA Netw. Open.

[29] House J.S., Mendez M., Maguire R.L., Gonzalez-Nahm S., Huang Z., Daniels J., Murphy S.K., Fuemmeler B.F., Wright F.A., Hoyo C. (2018). Periconceptional Maternal Mediterranean Diet Is Associated with Favorable Offspring Behaviors and Altered CpG Methylation of Imprinted Genes. Front. Cell Dev. Biol..

[30] Li Y.M., Shen Y.D., Li Y.J., Xun G.L., Liu H., Wu R.R., Xia K., Zhao J.P., Ou J.J. (2018). Maternal dietary patterns, supplements intake and autism spectrum disorders: A preliminary case-control study. Medicine.

[31] Gao L., Cui S.S., Han Y., Dai W., Su Y.Y., Zhang X. (2016). Does Periconceptional Fish Consumption by Parents Affect the Incidence of Autism Spectrum Disorder and Intelligence Deficiency? A Case-control Study in Tianjin, China. Biomed. Environ. Sci..

[32] Vecchione R., Wang S., Rando J., Chavarro J.E., Croen L.A., Fallin M.D., Hertz-Picciotto I., Newschaffer C.J., Schmidt R.J., Lyall K. (2022). Maternal Dietary Patterns during Pregnancy and Child Autism-Related Traits: Results from Two US Cohorts. Nutrients.

[33] Knapp E.A., Kress A.M., Parker C.B., Page G.P., McArthur K., Gachigi K.K., Alshawabkeh A.N., Aschner J.L., Bastain T.M., Breton C.V. (2023). The Environmental Influences on Child Health Outcomes (ECHO)-Wide Cohort. Am. J. Epidemiol..

[34] Bragg M.G., Westlake M., Alshawabkeh A.N., Bekelman T.A., Camargo C.A., Catellier D.J., Comstock S.S., Dabelea D., Dunlop A.L., Hedderson M.M. (2023). Opportunities for Examining Child Health Impacts of Early-Life Nutrition in the ECHO Program: Maternal and Child Dietary Intake Data from Pregnancy to Adolescence. Curr. Dev. Nutr..

[35] National Institute of Health (2024). Environmental Influences on Child Health Outcomes (ECHO) Program.

[36] Block G., Woods M., Potosky A., Clifford C. (1990). Validation of a self-administered diet history questionnaire using multiple diet records. J. Clin. Epidemiol..

[37] Willett W.C., Sampson L., Stampfer M.J., Rosner B., Bain C., Witschi J., Hennekens C.H., Speizer F.E. (1985). Reproducibility and validity of a semiquantitative food frequency questionnaire. Am. J. Epidemiol..

[38] National Cancer Institute (2010). National Institutes of Health, Epidemiology and Genomics Research Program.

[39] Subar A.F., Thompson F.E., Kipnis V., Midthune D., Hurwitz P., McNutt S., McIntosh A., Rosenfeld S. (2001). Comparative validation of the Block, Willett, and National Cancer Institute food frequency questionnaires: The Eating at America’s Table Study. Am. J. Epidemiol..

[40] Constantino J.N., Davis S.A., Todd R.D., Schindler M.K., Gross M.M., Brophy S.L., Metzger L.M., Shoushtari C.S., Splinter R., Reich W. (2003). Validation of a brief quantitative measure of autistic traits: Comparison of the social responsiveness scale with the autism diagnostic interview-revised. J. Autism Dev. Disord..

[41] Constantino J.N., Gruber C. (2005). Social Responsiveness Scale (SRS).

[42] Constantino J.N., Gruber C. (2012). Social Responsiveness Scale.

[43] Patti M.A., Croen L.A., Dickerson A.S., Joseph R.M., Ames J.L., Ladd-Acosta C., Ozonoff S., Schmidt R.J., Volk H.E., Hipwell A.E. (2024). Reproducibility between preschool and school-age Social Responsiveness Scale forms in the Environmental influences on Child Health Outcomes program. Autism Res..

[44] Sturm A., Kuhfeld M., Kasari C., McCracken J.T. (2017). Development and validation of an item response theory-based Social Responsiveness Scale short form. J. Child. Psychol. Psychiatry.

[45] Patti M.A., Ning X., Hosseini M., Croen L.A., Joseph R.M., Karagas M.R., Ladd-Acosta C., Landa R., Messinger D.S., Newschaffer C.J. (2023). A Comparative Analysis of the Full and Short Versions of the Social Responsiveness Scale in Estimating an Established Autism Risk Factor Association in ECHO: Do we Get the Same Estimates?. J. Autism Dev. Disord..

[46] Lyall K., Hosseini M., Ladd-Acosta C., Ning X., Catellier D., Constantino J.N., Croen L.A., Kaat A.J., Botteron K., Bush N.R. (2021). Distributional Properties and Criterion Validity of a Shortened Version of the Social Responsiveness Scale: Results from the ECHO Program and Implications for Social Communication Research. J. Autism Dev. Disord..

[47] Lyall K., Rando J., Toroni B., Ezeh T., Constantino J.N., Croen L.A., Garvin B., Piselli K., Connell J., Kaat A.J. (2022). Examining shortened versions of the Social Responsiveness Scale for use in autism spectrum disorder prediction and as a quantitative trait measure: Results from a validation study of 3–5 year old children. JCPP Adv..

[48] Koenker R., Hallock K.F. (2001). Quantile Regression. J. Econ. Perspect..

[49] Sandin S., Hultman C.M., Kolevzon A., Gross R., MacCabe J.H., Reichenberg A. (2012). Advancing maternal age is associated with increasing risk for autism: A review and meta-analysis. J. Am. Acad. Child. Adolesc. Psychiatry.

[50] Li Y.M., Ou J.J., Liu L., Zhang D., Zhao J.P., Tang S.Y. (2016). Association Between Maternal Obesity and Autism Spectrum Disorder in Offspring: A Meta-analysis. J. Autism Dev. Disord..

[51] Croen L.A., Grether J.K., Selvin S. (2002). Descriptive epidemiology of autism in a California population: Who is at risk?. J. Autism Dev. Disord..

[52] Hertz-Picciotto I., Korrick S.A., Ladd-Acosta C., Karagas M.R., Lyall K., Schmidt R.J., Dunlop A.L., Croen L.A., Dabelea D., Daniels J.L. (2022). Maternal tobacco smoking and offspring autism spectrum disorder or traits in ECHO cohorts. Autism Res..

[53] Maenner M.J., Warren Z., Williams A.R., Amoakohene E., Bakian A.V., Bilder D.A., Durkin M.S., Fitzgerald R.T., Furnier S.M., Hughes M.M. (2023). Prevalence and Characteristics of Autism Spectrum Disorder Among Children Aged 8 Years—Autism and Developmental Disabilities Monitoring Network, 11 Sites, United States, 2020. MMWR Surveill. Summ..

[54] Han V.X., Patel S., Jones H.F., Nielsen T.C., Mohammad S.S., Hofer M.J., Gold W., Brilot F., Lain S.J., Nassar N. (2021). Maternal acute and chronic inflammation in pregnancy is associated with common neurodevelopmental disorders: A systematic review. Transl. Psychiatry.

[55] Majerczyk D., Ayad E.G., Brewton K.L., Saing P., Hart P.C. (2022). Systemic maternal inflammation promotes ASD via IL-6 and IFN-γ. Biosci. Rep..

[56] Holingue C., Brucato M., Ladd-Acosta C., Hong X., Volk H., Mueller N.T., Wang X., Fallin M.D. (2020). Interaction between Maternal Immune Activation and Antibiotic Use during Pregnancy and Child Risk of Autism Spectrum Disorder. Autism Res..

[57] Millar S.R., Navarro P., Harrington J.M., Perry I.J., Phillips C.M. (2021). Dietary Quality Determined by the Healthy Eating Index-2015 and Biomarkers of Chronic Low-Grade Inflammation: A Cross-Sectional Analysis in Middle-to-Older Aged Adults. Nutrients.

[58] Fu Y., Tang L., Hu M., Xiang Z., Hu Y. (2020). Changes of serum interleukin-6 in healthy pregnant women and establishment of relevant reference intervals. Clin. Chim. Acta.

[59] Na X., Glasier C.M., Andres A., Ou X. (2023). Maternal Diet Quality during Pregnancy Is Associated with Neonatal Brain White Matter Development. Nutrients.

[60] Wolff J.J., Gu H., Gerig G., Elison J.T., Styner M., Gouttard S., Botteron K.N., Dager S.R., Dawson G., Estes A.M. (2012). Differences in white matter fiber tract development present from 6 to 24 months in infants with autism. Am. J. Psychiatry.

