# Supplementary material for: Maternal Dietary Patterns During Pregnancy and Child Autism-Related Traits in the Environmental Influences on Child Health Outcomes Consortium
Source: Nutrients. 2024 Nov 6;16(22):3802. doi: 10.3390/nu16223802 (PMC11597253; doi:10.3390/nu16223802)
Supplement: Supplementary file 1 [file nutrients-16-03802-s001.zip › nutrients-3244871-supplementary.pdf]

Figure S1: Study Participant Flowchart Across the Cohorts

Figure S2: Associations Between Dietary Patterns and SRS scores Removing One Cohort at a Time for: a) SRS:HEI, b) SRS:AHEIP, c)SRS:EDIP; d)ASD:HEI, e)ASD:AHEIP, f)ASD:EDIP)

Table S1: Diet Data Source by Cohort

Table S2: Participant Characteristics by Dietary Pattern Analytic Sample Among Those with SRS Outcome Data (SRS-Any n=4547, SRS-HEI n=2876, SRSAHEI-P n= 1671, SRS-EDIP n=2433)

Table S3: Participant Characteristics By Dietary Pattern Analytic Sample Among Those with ASD Outcome Data (ASD-Any n=5822, ASD-HEI n=4128, ASD-AHEI-P n= 1694, ASD-EDIP n=3614)

Table S4: Distribution (mean, SD) of Dietary Pattern Scores Across the Cohorts Included in Analytic Samples

Table S5: Association Between Maternal Dietary Patterns During Pregnancy and Child SRS Raw Scores Modeled Using Quantile Regression Fixed at the 10th, 20th, 70th, and 90th Percentiles

Table S6: Association Between Maternal Dietary Patterns During Pregnancy and Child SRS Raw Scores + Adjustment for Additional Factors

Table S7: Association Between Maternal Dietary Patterns During Pregnancy and Child ASD Diagnosis + Adjustment for Additional Factors

Table S8: Association Between Maternal Dietary Patterns During Pregnancy and Child SRS Raw Scores, Excluding SRS Preschool and Short Forms

Table S9: Association Between Maternal Dietary Patterns During Pregnancy and SRS T-scores

Figure S1: Study Participant Flowchart Across the Cohorts

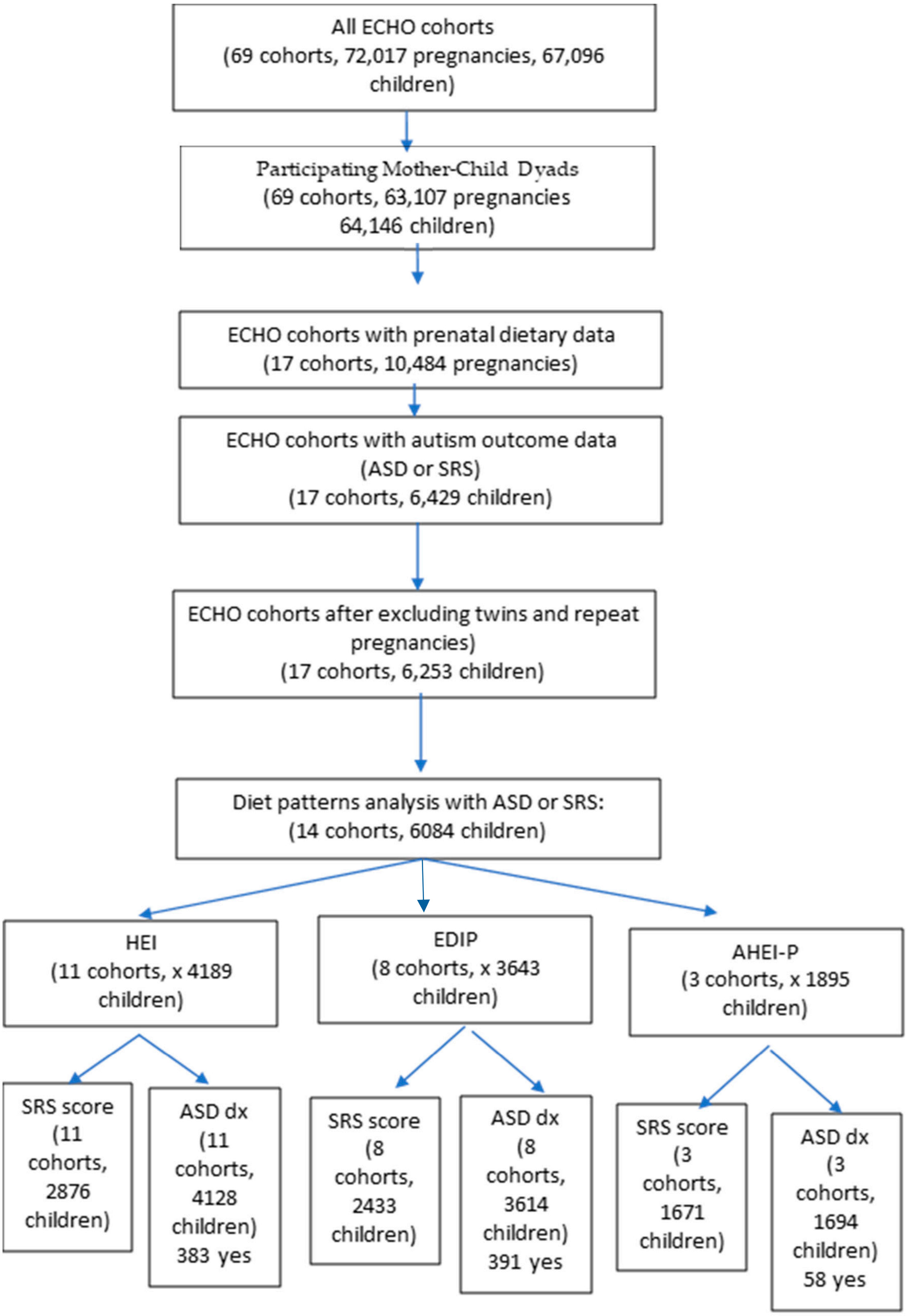

Figure S1:Several Cohorts contributed to multiple diet pattern analyses, and this can be seen further in the figures below

Figure S2: Associations Between Dietary Patterns and SRS scores Removing One Cohort at a Time for: a) SRS:HEI, b) SRS:AHEIP, c)SRS:EDIP; d)ASD:HEI, e)ASD:AHEIP, f)ASD:EDIP)

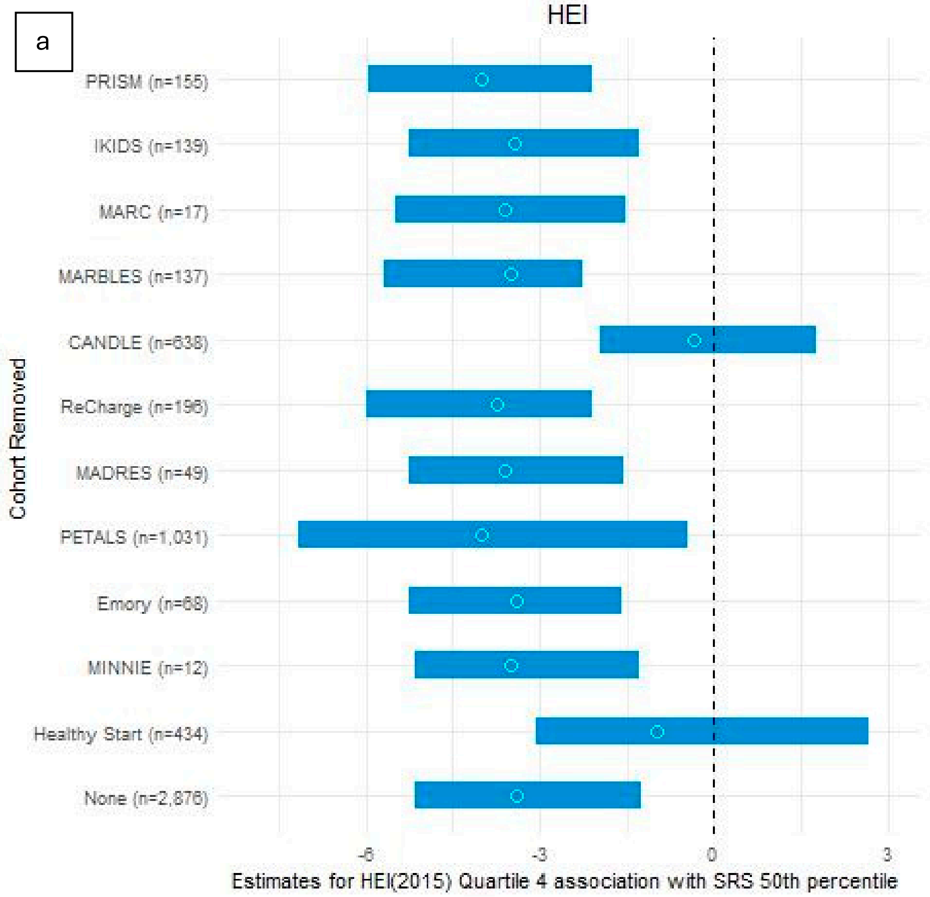

The indicated n values represent the number of participants in the listed cohort which was removed.

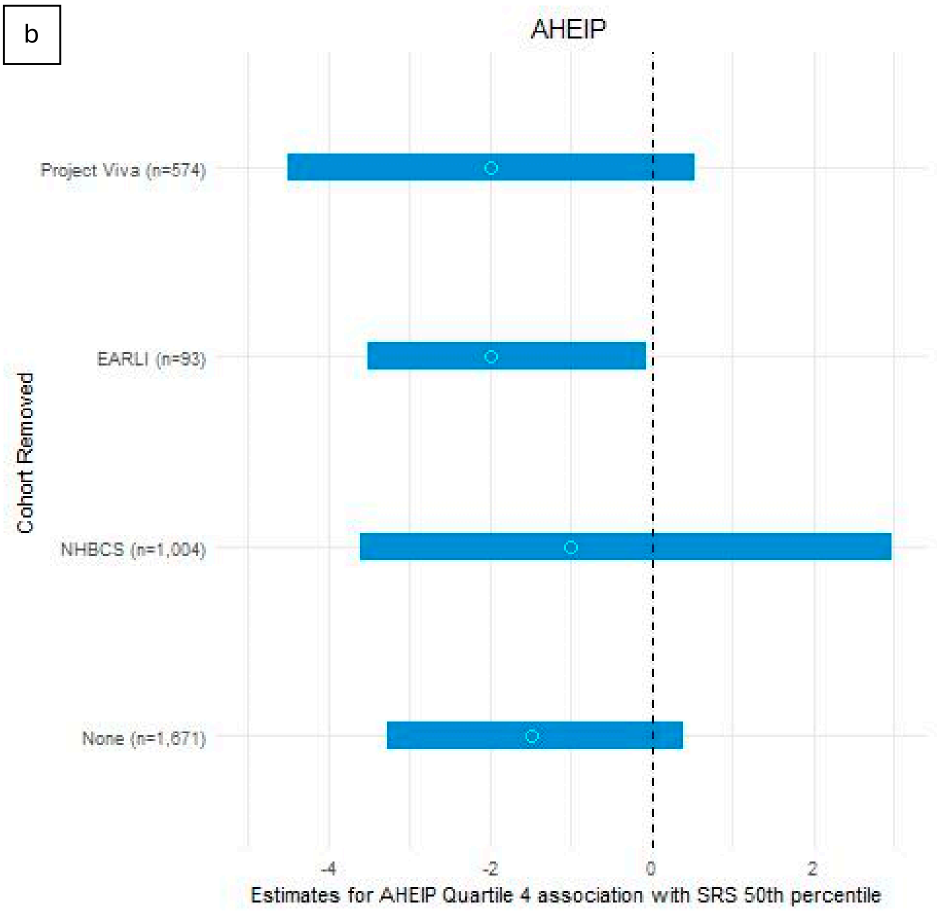

The indicated n values represent the number of participants in the listed cohort which was removed.

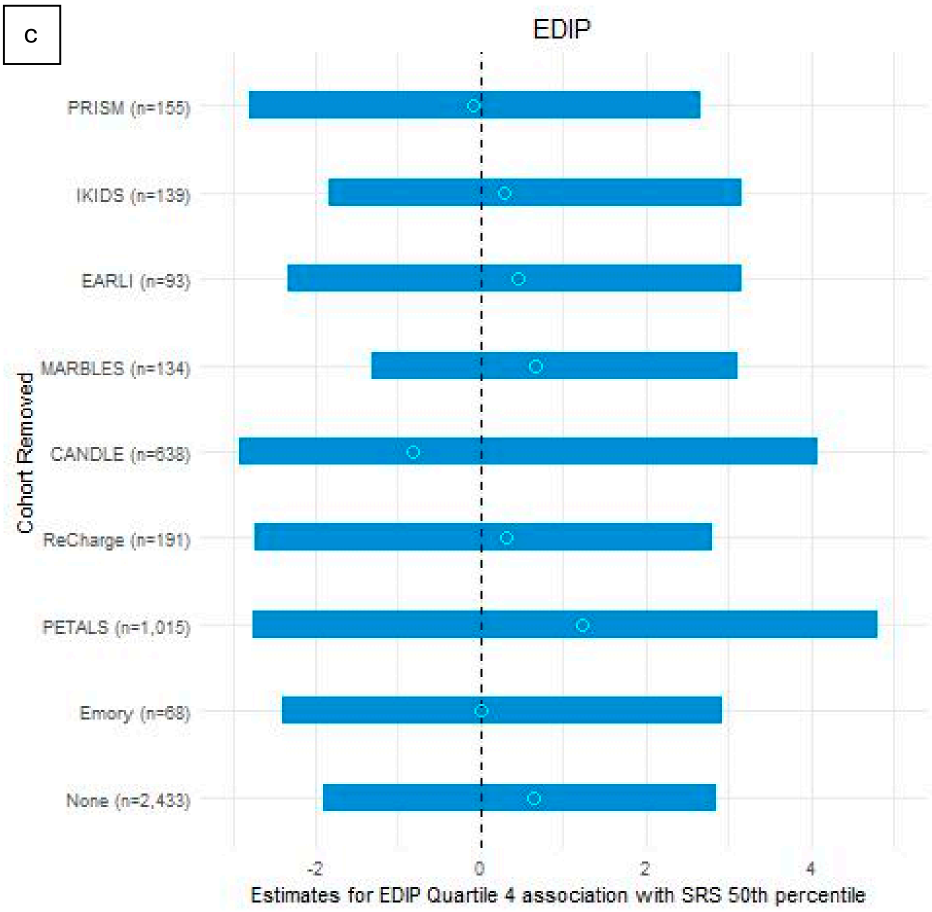

The indicated n values represent the number of participants in the listed cohort which was removed.

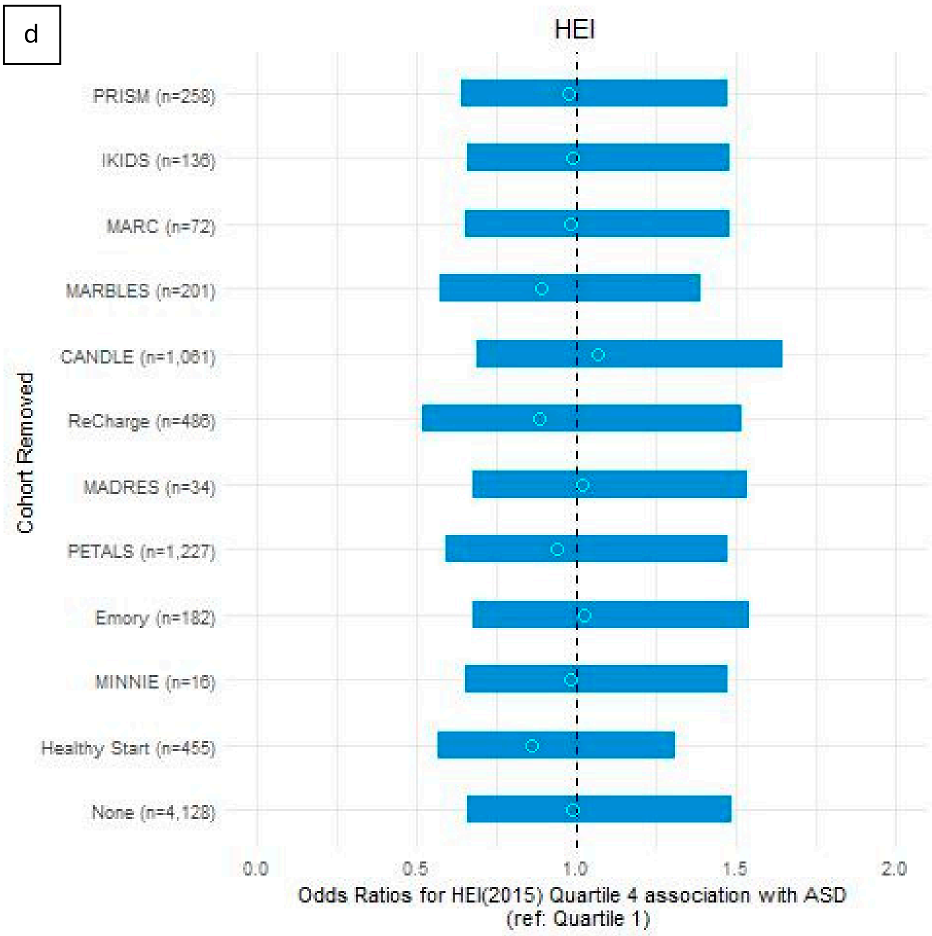

The indicated n values represent the number of participants in the listed cohort which was removed.

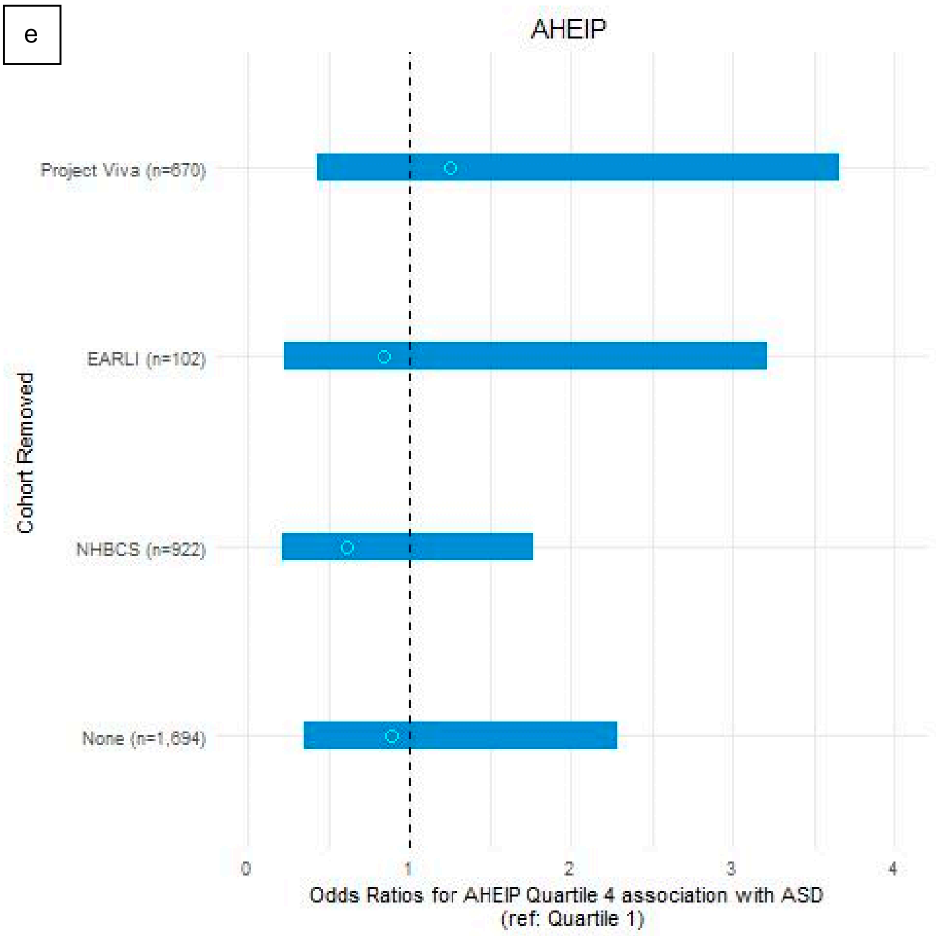

The indicated n values represent the number of participants in the listed cohort which was removed.

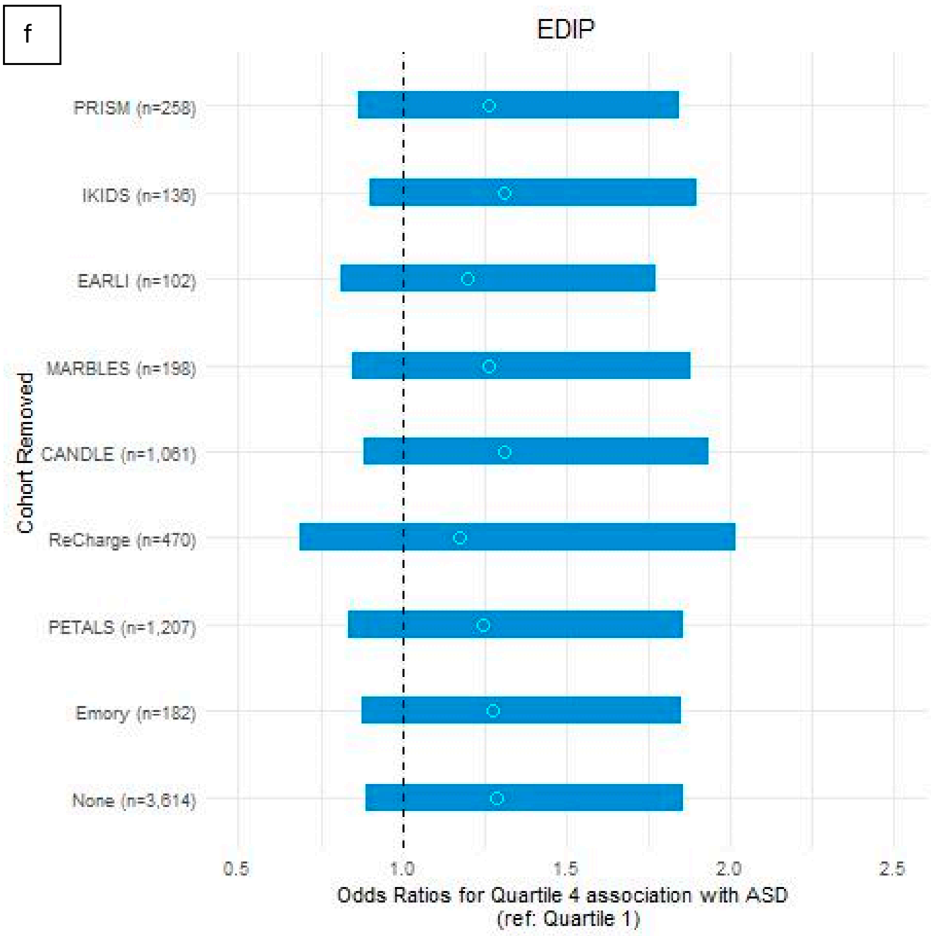

The indicated n values represent the number of participants in the listed cohort which was removed.

Table S1: Diet Data Source by Cohort

Cohort Name	Diet Source	HEI	EDIP	AHEI-P
Healthy Start	ASA-24	x		
Maternal Health Influences and Nutrition in Neonatal and Infant Development (MINNIE)	ASA-24	x		
Atlanta ECHO Cohort of Emory University	BLOCK	x	x	
Pregnancy Environment and Lifestyle Study (PETALS)	BLOCK	x	x	
Maternal and Developmental Risks from Environmental and Social Stressors (MADRES)	ASA-24	x		
Revisiting CHildhood Autism Risk from Genes and the Environment Study (ReCHARGE)	BLOCK	x	x	
New Hampshire Birth Cohort Study (NHBCS)	HARVARD FFQ			x
Conditions Affecting Neurocognitive Development and Learning in Early Childhood (CANDLE)	BLOCK	x	x	
University of California – Markers of Autism Risk in Babies (MARBLES)	BLOCK	x	x	
Early Autism Risk Longitudinal Investigation (EARLI)	DHQ2		x	x
Project Viva	HARVARD FFQ			x
Michigan Archive for Research in Child Health (MARCH)	BLOCK	x		
Illinois Kids Development Study (IKIDS)	BLOCK	x	x	
PRogramming of Intergenerational Stress Mechanisms (PRISM)	BLOCK	x	x	

Table S2: Participant Characteristics by Dietary Pattern Analytic Sample Among Those with SRS Outcome Data (SRS-Any n=4547, SRS-HEI n=2876, SRSAHEI-P n= 1671, SRS-EDIP n=2433)

Characteristics	SRS-Any n(%)	SRS-HEI n(%)	SRS-AHEI-P n(%)	SRS-EDIP n(%)
Cohort Type				
High Familial Likelihood	230(5%)	137(5%)	93(6%)	227(9%)
Population based	4,317(95%)	2,739(95%)	1,578(94%)	2,206(91%)
Maternal and Child Characteristics				
Maternal race				
Asian & Pacific Islander	380(8%)	324(11%)	56(3%)	317(13%)
Black/African American	728(16%)	612(21%)	116(7%)	564(23%)
Native American or Native Alaskan	27(1%)	20(1%)	7(0%)	17(1%)
White	2,979(66%)	1,540(54%)	1,439(86%)	1,169(48%)
Multiple/Other Race	276(6%)	230(8%)	46(3%)	215(9%)
Unknown/missing	157(3%)	150(5%)	7(0%)	151(6%)
Maternal ethnicity				
Hispanic/Latino	763(17%)	680(24%)	>80(>5%)	560(23%)
Not Hispanic/Latino	3,771(83%)	2,188(76%)	1,583(95%)	1,866(77%)
Missing	13(0%)	8(0%)	<5	7(0%)
Maternal age, years				
<18-28 years	1,331(29%)	1,012(35%)	319(19%)	843(35%)
29-34 years	1,992(44%)	1,198(42%)	794(48%)	990(41%)
35-40 years	1,087(24%)	602(21%)	485(29%)	540(22%)
41+ years	137(3%)	64(2%)	73(4%)	60(2%)
Maternal education				
Less than high school	183(4%)	166(6%)	17(1%)	123(5%)
HS degree, GED or equivalent	617(14%)	496(17%)	121(7%)	439(18%)
Some college, no degree, Assoc/Trade	1,021(22%)	688(24%)	333(20%)	600(25%)
Bachelor's degree (BA, BS)	1,426(31%)	842(29%)	584(35%)	722(30%)
Masters, Prof or Doctorate Degree	1,235(27%)	<685(>24%)	556(33%)	>545(>22%)
Missing	65(1%)	<5	60(4%)	<5
Pre-Pregnancy BMI, kg/m2				
<18.5	118(3%)	71(2%)	47(3%)	66(3%)
18.5-24.9	2,163(48%)	1,232(43%)	931(56%)	1,007(41%)
25-29.9	1,178(26%)	767(27%)	411(25%)	644(26%)
>=30	1,065(23%)	789(27%)	276(17%)	700(29%)

Missing	23(1%)	17(1%)	6(0%)	16(1%)
Prenatal smoking				
Active	206(5%)	135(5%)	71(4%)	111(5%)
Not active	4,315(95%)	2,734(95%)	1,581(95%)	2,308(95%)
Missing	26(1%)	7(0%)	19(1%)	14(1%)
Ever breastfeed				
Yes	3,284(72%)	2,073(72%)	>1,210(>72%)	1,680(69%)
No	63(1%)	60(2%)	<5	43(2%)
Missing	1,200(26%)	743(26%)	457(27%)	710(29%)
Prenatal vitamin use				
Yes	1,925(42%)	1,490(52%)	N/A	1,045(43%)
No	114(3%)	91(3%)	23(1%)	85(3%)
Missing	2,508(55%)	1,295(45%)	1,213(73%)	1,303(54%)
Prenatal vitamin use (first month)				
Yes	274(6%)	274(10%)	1,671(100%)	<5
No	174(4%)	174(6%)	435(26%)	<5
Missing	4,099(90%)	2,428(84%)	N/A	2,430(100%)
Child sex				
Male	2,357(52%)	1,535(53%)	822(49%)	1,320(54%)
Female	2,190(48%)	1,341(47%)	849(51%)	1,113(46%)
Child year of birth				
1999-2004	574(13%)	N/A	574(34%)	N/A
2005-2009	436(10%)	385(13%)	51(3%)	385(16%)
2010-2014	2,042(45%)	1,229(43%)	813(49%)	879(36%)
2015+	1,495(33%)	1,262(44%)	233(14%)	1,169(48%)
Birthweight				
Small for gestational age	223(5%)	170(6%)	53(3%)	139(6%)
Normal for gestational age	3,417(75%)	2,189(76%)	1,228(73%)	1,820(75%)
Large for gestational age	750(16%)	391(14%)	359(21%)	357(15%)
Missing	157(3%)	126(4%)	31(2%)	117(5%)
ASD diagnosis				
Yes	252(6%)	200(7%)	52(3%)	215(9%)
No	4,033(89%)	2,615(91%)	1,418(85%)	2,189(90%)
ASD not available	262(6%)	61(2%)	201(12%)	29(1%)
Total SRS raw score				
SRS available	4,547(100%)	2,876(100%)	1,671(100%)	2,433(100%)
	**Mean (Std)**			
Parity	0.83(0.96)	0.83(0.97)	0.83(0.94)	0.84(0.96)
Total caloric intake, kcal	2,004(945)	1,951(1,045)	2,095(733)	1,925(1,090)
Diet Score	N/A	64.3 (12.7)	58.0 (12.1)	0.42 (0.52)
SRS Score	29.3(22.5)	30.9(24.0)	26.5(19.1)	31.5(25.1)

Table S3: Participant Characteristics By Dietary Pattern Analytic Sample Among Those with ASD Outcome Data (ASD-Any n=5822, ASD-HEI n=4128, ASD-AHEI-P n= 1694, ASD-EDIP n=3614)

Characteristics	ASD-Any n(%)	ASD-HEI n(%)	ASD-AHEI-P n(%)	ASD-EDIP n(%)
Cohort Type				
High Familial Likelihood	303(5%)	201(5%)	102(6%)	300(8%)
Population based	5,519(95%)	3,927(95%)	1,592(94%)	3,314(92%)
Maternal and Child Characteristics				
Maternal race				
Asian & Pacific Islander	487(8%)	424(10%)	63(4%)	411(11%)
Black/African American	1,208(21%)	1,087(26%)	121(7%)	1,026(28%)
Native American or Native Alaskan	30(1%)	22(1%)	8(0%)	20(1%)
White	3,498(60%)	2,049(50%)	1,449(86%)	1,626(45%)
Multiple/Other Race	364(6%)	317(8%)	47(3%)	302(8%)
Unknown/missing	235(4%)	229(6%)	6(0%)	229(6%)
Maternal ethnicity				
Hispanic/Latino	991(17%)	898(22%)	>90(>5%)	787(22%)
Not Hispanic/Latino	4,813(83%)	3,217(78%)	1,596(94%)	2,816(78%)
Missing	18(0%)	13(0%)	<5	11(0%)
Maternal age, years				
<18-28 years	1,910(33%)	1,572(38%)	338(20%)	1,386(38%)
29-34 years	2,441(42%)	1,644(40%)	797(47%)	1,406(39%)
35-40 years	1,305(22%)	816(20%)	489(29%)	732(20%)
41+ years	166(3%)	96(2%)	70(4%)	90(2%)
Maternal education				
Less than high school	300(5%)	280(7%)	20(1%)	235(7%)
HS degree, GED or equivalent	975(17%)	840(20%)	135(8%)	778(22%)
Some college, no degree, Assoc/Trade	1,371(24%)	1,002(24%)	369(22%)	892(25%)
Bachelor's degree (BA, BS)	1,698(29%)	1,131(27%)	567(33%)	997(28%)
Masters, Prof or Doctorate Degree	1,414(24%)	863(21%)	551(33%)	703(19%)
Missing	64(1%)	12(0%)	52(3%)	9(0%)
Pre-Pregnancy BMI, kg/m2				
<18.5	168(3%)	117(3%)	51(3%)	110(3%)
18.5-24.9	2,695(46%)	1,762(43%)	933(55%)	1,500(42%)
25-29.9	1,490(26%)	1,068(26%)	422(25%)	929(26%)
>=30	1,408(24%)	1,128(27%)	280(17%)	1,029(28%)

Missing	61(1%)	53(1%)	8(0%)	46(1%)
Prenatal smoking				
Active	305(5%)	231(6%)	74(4%)	200(6%)
Not active	5,490(94%)	3,890(94%)	1,600(94%)	3,397(94%)
Missing	27(0%)	7(0%)	20(1%)	17(0%)
Ever breastfeed				
Yes	4,035(69%)	2,850(69%)	>1,190(>70%)	2,386(66%)
No	114(2%)	111(3%)	<5	90(2%)
Missing	1,673(29%)	1,167(28%)	506(30%)	1,138(31%)
Prenatal vitamin use				
Yes	2,913(50%)	2,406(58%)	N/A	1,926(53%)
No	175(3%)	145(4%)	30(2%)	138(4%)
Missing	2,734(47%)	1,577(38%)	1,157(68%)	1,550(43%)
Prenatal vitamin use (first month)				
Yes	289(5%)	289(7%)	1,694(100%)	N/A
No	181(3%)	181(4%)	507(30%)	<5
Missing	5,352(92%)	3,658(89%)	N/A	>3,610(<100%)
Child sex				
Male	3,057(53%)	2,229(54%)	828(49%)	1,977(55%)
Female	2,765(47%)	1,899(46%)	866(51%)	1,637(45%)
Child year of birth				
1999-2004	670(12%)	N/A	670(40%)	N/A
2005-2009	802(14%)	750(18%)	52(3%)	747(21%)
2010-2014	2,560(44%)	1,771(43%)	789(47%)	1,400(39%)
2015+	1,790(31%)	1,607(39%)	183(11%)	1,467(41%)
Birthweight				
Small for gestational age	311(5%)	263(6%)	48(3%)	229(6%)
Normal for gestational age	4,387(75%)	3,133(76%)	1,254(74%)	2,707(75%)
Large for gestational age	955(16%)	581(14%)	374(22%)	536(15%)
Missing	169(3%)	151(4%)	18(1%)	142(4%)
ASD diagnosis				
Yes	441(8%)	383(9%)	58(3%)	391(11%)
No	5,381(92%)	3,745(91%)	1,636(97%)	3,223(89%)
Total SRS raw score				
SRS not available	1,537(26%)	1,313(32%)	224(13%)	1,210(33%)
SRS available	4,285(74%)	2,815(68%)	1,470(87%)	2,404(67%)
	**Mean (Std)**			
Parity	0.83(0.98)	0.85(1.00)	0.82(0.94)	0.85(1.00)
Total caloric intake, kcal	2,060(1,058)	2,053(1,166)	2,079(733)	2,051(1,222)
Diet Score	N/A	64.2 (12.3)	58.1 (12.0)	0.47 (0.59)
SRS Score	29.2(22.4)	30.6(23.7)	26.4(19.4)	31.4(25.0)

Table S4: Distribution (mean, SD) of Dietary Pattern Scores Across the Cohorts Included in Analytic Samples

Cohort Name	Analytic Sample (n)	HEI	EDIP	AHEIP
Healthy Start	ASD+SRS 468<br>ASD 455<br>SRS 434	49.65 (9.95)<br>49.69 (9.92)<br>49.71 (10.06)		
Maternal Health Influences and Nutrition in Neonatal and Infant Development (MINNIE)	ASD+SRS 17<br>ASD 16<br>SRS 12	47.62 (12.57)<br>47.94 (12.91)<br>49.58 (14.22)		
Atlanta ECHO Cohort of Emory University	ASD+SRS 182 182<br>ASD 182 182<br>SRS 68 68	60.71 (9.39)<br>60.71 (9.39)<br>63.29 (9.40)	0.56 (0.52)<br>0.56 (0.52)<br>0.47 (0.45)	
Pregnancy Environment and Lifestyle Study (PETALS)	ASD+SRS 1227 1207<br>ASD 1227 1207<br>SRS 1031 1015	71.89 (9.34)<br>71.89 (9.34)<br>72.01 (9.24)	0.29 (0.35)<br>0.29 (0.35)<br>0.29 (0.34)	
Maternal and Developmental Risks from Environmental and Social Stressors (MADRES)	ASD+SRS 49<br>ASD 34<br>SRS 49	44.90 (9.60)<br>43.50 (9.95)<br>44.90 (9.60)		
Revisiting CHildhood Autism Risk from Genes and the Environment Study (ReCHARGE)	ASD+SRS 486 470<br>ASD 486 470<br>SRS 196 191	66.69 (9.43)<br>66.69 (9.43)<br>67.15 (9.09)	0.60 (0.64)<br>0.60 (0.64)<br>0.57 (0.51)	
New Hampshire Birth Cohort Study (NHBCS)	ASD+SRS 1121<br>ASD 922<br>SRS 1004			56.67 (12.46)<br>56.31 (12.49)<br>56.66 (12.48)
Conditions Affecting Neurocognitive Development and Learning in Early Childhood (CANDLE)	ASD+SRS 1062 1062<br>ASD 1061 1061<br>SRS 638 638	62.29 (10.34)<br>62.30 (10.34)<br>62.34 (10.34)	0.69 (0.76)<br>0.69 (0.76)<br>0.66 (0.73)	

University of California – Markers of Autism Risk in Babies (MARBLES)	ASD+SRS<br>ASD 201 198<br>SRS 137 134	66.11 (9.71)<br>66.13 (9.65)<br>66.30 (9.81)	0.31 (0.31)<br>0.31 (0.31)<br>0.32 (0.30)	
Early Autism Risk Longitudinal Investigation (EARLI)	ASD+SRS 103 103<br>ASD 102 102<br>SRS 93 93		0.14 (0.35)<br>0.14 (0.35)<br>0.17 (0.31)	52.72 (14.10)<br>52.78 (14.16)<br>52.17 (14.08)
Project Viva	ASD+SRS 671<br>ASD 670<br>SRS 574			61.35 (9.96)<br>61.32 (9.94)<br>61.32 (9.96)
Michigan Archive for Research in Child Health (MARCH)	ASD+SRS 76<br>ASD 72<br>SRS 17	47.80 (14.35)<br>48.29 (14.26)<br>44.36 (13.98)		
Illinois Kids Development Study (IKIDS)	ASD+SRS 141 141<br>ASD 136 136<br>SRS 139 139	64.82 (9.86)<br>64.82 (9.88)<br>64.80 (9.86)	0.19 (0.30)<br>0.18 (0.31)<br>0.19 (0.30)	
PRogramming of Intergenerational Stress Mechanisms (PRISM)	ASD+SRS 275 275<br>ASD 258 258<br>SRS 155 155	65.00 (11.30)<br>65.05 (11.07)<br>66.59 (10.75)	0.52 (0.55)<br>0.53 (0.55)<br>0.47 (0.44)	

Table S5: Association Between Maternal Dietary Patterns During Pregnancy and Child SRS Raw Scores Modeled Using Quantile Regression Fixed at the 10th, 20th, 70th, and 90th Percentiles

Percentile	Quartile	HEI	AHEI-P	EDIP
		Adjusted (ß, 95% CI)	Adjusted (ß, 95% CI	Adjusted (ß, 95% CI
0.1	Q1	0 (reference)	0 (reference)	0 (reference)
	Q2	-1.60 (-2.86,-0.70)	-2.00 (-4.22,-0.42)	-1.00 (-2.12,0.24)
	Q3	-3.40 (-4.75,-2.05)	-2.00 (-4.62,0.37)	-1.00 (-1.97,0.03)
	Q4	-1.80 (-3.31,-0.55)	-2.00 (-3.90,-0.48)	-1.50 (-2.24,-0.11)
0.2	Q1	0 (reference)	0 (reference)	0 (reference)
	Q2	-1.33 (-3.11,-0.16)	-2.33 (-4.00,-0.51)	-1.25 (-2.87,0.30)
	Q3	-3.33 (-5.21,-2.76)	-2.33 (-4.43,-0.61)	-0.75 (-2.23,0.71)
	Q4	-3.33 (-5.35,-2.23)	-2.00 (-4.35,-0.74)	-0.13 (-1.81,1.49)
0.7	Q1	0 (reference)	0 (reference)	0 (reference)
	Q2	-4.00 (-7.98,-0.30)	0.00 (-2.76,3.68)	-2.43 (-4.08,1.96)
	Q3	-5.00 (-8.51,-1.05)	-0.50 (-3.85,3.59)	-2.52 (-4.40,1.22)
	Q4	-5.00 (-8.44,-1.82)	-1.50 (-5.09,2.69)	1.86 (-1.40,5.97)
0.9	Q1	0 (reference)	0 (reference)	0 (reference)
	Q2	-0.00 (-5.51,5.36)	-1.83 (-7.57,4.94)	-0.50 (-4.52,2.63)
	Q3	-2.50 (-7.99,2.92)	-2.50 (-10.15,3.85)	0.70 (-4.99,3.98)
	Q4	-4.50 (-9.48,0.69)	-4.00 (-12.46,2.24)	2.40 (-2.87,7.87)

Modeled using quantile regression fixed at indicated percentile of SRS total raw score
Adjusted: maternal age, maternal pre-pregnancy BMI, child sex (male, female), cohort type (familial/not-assign Recharge to ASD High group), maternal ethnicity/race (non-Hispanic white, non-Hispanic Black, Hispanic, Other), maternal education (less than high school, high school/GED, some college/associates degree/trade school, bachelor's degree, graduate degree), maternal smoking (yes, no), and child year of birth (1998-2004, 2005-2009, 2010-2014, 2015+)

Table S6: Association Between Maternal Dietary Patterns During Pregnancy and Child SRS Raw Scores + Adjustment for Additional Factors

	Adjusted (PRIMARY RESULT) **(ß, 95% CI)**	**Adjusted + diet form** type **(ß, 95% CI)**	**Adjusted + supplement use (ß, 95% CI)**	**Adjusted + dietary folic acid (ß, 95% CI)**	**Adjusted + parity (ß, 95% CI)**	**Adjusted + breastfeeding Ever (ß, 95% CI)**	**Adjusted + Income (ß, 95% CI)**	**Adjusted + Preterm (ß, 95% CI)**	**Adjusted + Birth size (ß, 95% CI)**	**Adjusted + GDB (ß, 95% CI)**
EDIP	**n= 2433**	**n= 2433**	**n= 1130**	**n=2431**	**n= 2433**	**n= 2433**	**n= 2433**	**n= 2433**	**n= 2433**	**n= 2433**
Q1	0 (reference)	0 (reference)	0 (reference)	0 (reference)	0 (reference)	0 (reference)	0 (reference)	0 (reference)	0 (reference)	0 (reference)
Q2	-1.25 (-3.73,0.65)	-1.45 (-3.74,0.86)	-0.00 (-5.21,3.31)	-1.34 (-3.82, 0.73)	-1.71 (-3.97,0.44)	-1.36 (-3.88,1.16)	-1.26 (-3.85,1.34)	-1.25 (-3.90,1.03)	-1.38 (-3.81,1.06)	-1.13 (-3.50,0.91)
Q3	-0.42 (-3.44,2.11)	-0.55 (-3.25,2.17)	0.00 (-4.85,2.81)	-0.46 (-3.51,2.26)	-0.08 (-2.34,2.04)	-1.14 (-3.31,1.04)	-0.58 (-2.78,1.61)	-0.92 (-3.44,2.37)	-0.50 (-2.93,1.93)	-0.80 (-3.39,1.93)
Q4	0.64 (-1.91,2.85)	0.36 (-2.20,2.99)	3.00 (-1.60,5.57)	0.51 (-2.57,2.59)	0.90 (-1.99,3.74)	-0.09 (-2.71,2.53)	0.45 (-2.04,2.95)	0.33 (-2.12,2.96)	0.50 (-2.15,3.15)	0.40 (-1.99,2.99)
AHEIP	**n= 1671**	**n= 1671[a]**	**n= 458[b]**	**n= 1669**	**n= 1187**	**n= 1671**	**n= 101**	**n= 1671**	**n= 1671**	**n= 1671**
Q1	0 (reference)	0 (reference)	0 (reference)	0 (reference)	0 (reference)	0 (reference)	-	0 (reference)	0 (reference)	0 (reference)
Q2	0.00 (-2.56,1.79)	0.00 (-2.41,1.79)	-3.00 (-7.57,1.97)	0.06 (-2.23,2.35)	-0.33 (-3.32,2.65)	0.00 (-2.31,2.31)	-	0.00 (-2.22,2.22)	0.00 (-2.13,2.13)	-0.00 (-2.09,2.09)
Q3	-1.50 (-3.95,0.86)	-1.50 (-3.95,0.90)	-3.00 (-6.00,1.94)	-1.85 (-4.49, 0.78)	-0.33 (-3.53,2.86)	-1.50 (-4.22,1.22)	-	-1.57 (-3.99,0.85)	-1.00 (-3.32,1.32)	-1.50 (-4.12,1.12)
Q4	-1.50 (-3.27,0.39)	-1.50 (-3.45,0.39)	-1.00 (-3.90,3.41)	-2.12 (-4.28, 0.04)	-0.33 (-3.16,2.49)	-1.50 (-3.85,0.85)	-	-1.57 (-3.71,0.57)	-1.67 (-3.83,0.50)	-1.50 (-3.70,0.70)
HEI	**n= 2876**	**n= 2876**	**n= 2876**	**n=2876**	**n= 2876**	**n= 2876**	**n= 2876**	**n= 2876**	**n= 2876**	**n= 2876**
Q1	0 (reference)	0 (reference)	0 (reference)	0 (reference)	0 (reference)	0 (reference)	0 (reference)	0 (reference)	0 (reference)	0 (reference)
Q2	-1.84 (-3.99,-0.20)	-0.33 (-2.12,1.86)	-2.42 (-4.36,-0.48)	-1.82 (-4.18, -0.25)	-2.20 (-4.98,0.07)	-0.47 (-2.30,1.36)	-2.25 (-4.34,-0.16)	-1.80 (-3.98,-0.03)	-2.07 (-4.01,-0.13)	-2.03 (-3.68,0.01)
Q3	-4.24 (-6.17,-2.26)	-2.00 (-3.79,0.46)	-4.62 (-6.80,-2.45)	-4.13 (-6.18,-2.24)	-4.21 (-6.21,-1.83)	-2.35 (-4.61,-0.10)	-4.75 (-6.75,-2.75)	-4.20 (-6.09,-2.12)	-4.47 (-6.41,-2.53)	-4.03 (-5.58,-1.86)

Q4	-3.41 (-5.15,-1.26)	-1.00 (-3.22,2.02)	-4.79 (-7.02,-2.56)	-3.28 (-5.15,-1.13)	-3.15 (-5.93,-1.03)	-2.00 (-4.04,0.04)	-4.00 (-6.28,-1.72)	-3.40 (-5.22,-1.15)	-3.24 (-5.37,-1.12)	-3.55 (-4.97,-1.05)

Modeled using quantile regression fixed at 50th percentile

Adjusted: maternal age, maternal pre-pregnancy BMI, child sex (male, female), cohort type (familial/not-assign Recharge to ASD High group), maternal ethnicity/race (non-Hispanic white, non-Hispanic Black, Hispanic, Other), maternal education (less than high school, high school/GED, some college/associates degree/trade school, bachelor's degree, graduate degree), maternal smoking (yes, no), and child year of birth (1998-2004, 2005-2009, 2010-2014, 2015+)

[a]For this model cohort type was not included in adjusted models due to collinearity with form type

[b]This model was run in a subset due to data availability

*Supplement use in the 1st month could not be analyzed due to high missingness

Table S7: Association Between Maternal Dietary Patterns During Pregnancy and Child ASD Diagnosis + Adjustment for Additional Factors

	Adjusted (OR, 95% CI)	Adjusted + form (OR, 95% CI)	Adjusted + supplement use (OR, 95% CI)	Adjusted + dietary folic acid (OR, 95% CI)	Adjusted + parity (OR, 95% CI)	Adjusted + breastfeeding Ever (OR, 95% CI)	Adjusted + Income (OR, 95% CI)	Adjusted + Preterm (OR, 95% CI)	Adjusted + Birth size (OR, 95% CI)	Adjusted + GDB (OR, 95% CI)
EDIP	**n= 3614**	**n= 3614**	**n= 3614**	**n= 3614**	**n= 3614**	**n= 3614**	**n= 3614**	**n= 3614**	**n= 3614**	**n= 3614**
Q1	1 (reference)	1 (reference)	1 (reference)	1 (reference)	1 (reference)	1 (reference)	1 (reference)	1 (reference)	1 (reference)	1 (reference)
Q2	1.27 (0.88,1.82)	1.19 (0.82,1.71)	1.24 (0.86,1.78)	1.27 (0.88,1.82)	1.27 (0.88,1.84)	1.26 (0.87,1.81)	1.23 (0.85,1.78)	1.27 (0.88,1.82)	1.27 (0.88,1.84)	1.25 (0.87,1.80)
Q3	1.27 (0.89,1.81)	1.18 (0.83,1.69)	1.23 (0.86,1.75)	1.27 (0.89,1.81)	1.28 (0.90,1.83)	1.26 (0.89,1.80)	1.24 (0.87,1.77)	1.27 (0.89,1.81)	1.28 (0.90,1.82)	1.27 (0.89,1.81)
Q4	1.29 (0.89,1.86)	1.16 (0.80,1.70)	1.22 (0.84,1.78)	1.29 (0.89,1.86)	1.28 (0.88,1.86)	1.29 (0.89,1.86)	1.24 (0.85,1.79)	1.28 (0.89,1.86)	1.30 (0.90,1.89)	1.28 (0.89,1.85)
AHEI-P	**n= 1694**	**n= 1694[a]**	**n= 537[b]**	**n= 1694**	**n= 1246**	**n= 1694**	**n= 111**	**n= 1694**	**n= 1694**	**n= 1694**
Q1	1 (reference)	1 (reference)	1 (reference)	1 (reference)	1 (reference)	1 (reference)	-	1 (reference)	1 (reference)	1 (reference)
Q2	1.14 (0.49,2.65)	1.14 (0.49,2.65)	0.90 (0.15,5.33)	1.14 (0.49, 2.65)	1.10 (0.46,2.67)	1.32 (0.55,3.15)	-	1.14 (0.49,2.64)	1.16 (0.50,2.71)	1.14 (0.49,2.64)
Q3	1.23 (0.52,2.90)	1.23 (0.52,2.90)	1.01 (0.18,5.61)	1.23 (0.52, 2.89)	1.25 (0.51,3.08)	1.39 (0.57,3.36)	-	1.24 (0.53,2.93)	1.24 (0.52,2.92)	1.21 (0.51,2.87)
Q4	0.89 (0.35,2.28)	0.89 (0.35,2.28)	0.50 (0.08,3.35)	0.89 (0.35, 2.28)	0.82 (0.31,2.21)	0.99 (0.38,2.59)	-	0.91 (0.35,2.34)	0.89 (0.34,2.29)	0.89 (0.35,2.29)
HEI	**n= 4128**	**n= 4128**	**n= 4128**	**n= 4128**	**n= 4128**	**n= 4128**	**n= 4128**	**n= 4128**	**n= 4128**	**n= 4128**
Q1	1 (reference)	1 (reference)	1 (reference)	1 (reference)	1 (reference)	1 (reference)	1 (reference)	1 (reference)	1 (reference)	1 (reference)

Q2	1.04 (0.71,1.51)	0.93 (0.63,1.38)	1.01 (0.69,1.48)	1.04 (0.71, 1.51)	1.04 (0.71,1.52)	1.04 (0.72,1.52)	1.02 (0.70,1.48)	1.04 (0.71,1.51)	1.03 (0.71,1.50)	1.03 (0.71,1.50)
Q3	0.92 (0.62,1.35)	0.81 (0.54,1.22)	0.89 (0.60,1.31)	0.92 (0.62, 1.35)	0.91 (0.61,1.35)	0.93 (0.63,1.38)	0.90 (0.61,1.33)	0.92 (0.62,1.35)	0.91 (0.61,1.34)	0.92 (0.62,1.35)
Q4	0.99 (0.66,1.49)	0.87 (0.57,1.32)	0.96 (0.63,1.44)	0.99 (0.66, 1.49)	0.94 (0.62,1.42)	1.01 (0.67,1.51)	0.97 (0.64,1.46)	0.99 (0.66,1.49)	0.98 (0.65,1.47)	0.98 (0.66,1.48)

Adjusted: maternal age, maternal pre-pregnancy BMI, child sex (male, female), cohort type (familial/not-assign Recharge to ASD High group), maternal ethnicity/race (non-Hispanic white, non-Hispanic Black, Hispanic, Other), maternal education (less than high school, high school/GED, some college/associates degree/trade school, bachelor’s degree, graduate degree), maternal smoking (yes, no), and child year of birth (1998-2004, 2005-2009, 2010-2014, 2015+)

[a]For this model cohort type was not included in adjusted models due to collinearity with form type

[b]This model was run in a subset due to data availability

*Supplement use in the 1st month could not be analyzed due to high missingness

Table S8: Association Between Maternal Dietary Patterns During Pregnancy and Child SRS Raw Scores, Excluding SRS Preschool and Short Forms

	**Primary result: Adjusted (ß, 95% CI)**	**Adjusted (ß, 95% CI) excluding SRS preschool and short forms**
EDIP	n= 2433	n=1765
Q1	0 (reference)	0 (reference)
Q2	-1.25 (-3.73,0.65)	-0.78 (-1.98,2.77)
Q3	-0.42 (-3.44,2.11)	0.11 (-1.92,2.89)
Q4	0.64 (-1.91,2.85)	0.67 (-1.66,4.75)
AHEI-P	n =1671	n=1318
Q1	0 (reference)	0 (reference)
Q2	0.00 (-2.56,1.79)	-0.38 (-2.84,0.99)
Q3	-1.50 (-3.95,0.86)	-1.38 (-3.40,1.66)
Q4	-1.50 (-3.27,0.39)	-1.38 (-3.28,0.75)
HEI	n= 2876	n= 2229
Q1	0 (reference)	0 (reference)
Q2	-1.84 (-3.99,-0.20)	-0.00 (-3.01,1.98)
Q3	-4.24 (-6.17,-2.26)	-2.00 (-4.97,1.58)
Q4	-3.41 (-5.15,-1.26)	-1.00 (-3.66,1.00)

SRS results modeled using quantile regression fixed at 50th percentile
Adjusted: maternal age, maternal pre-pregnancy BMI, child sex (male, female), cohort type (familial/not-assign Recharge to ASD High group), maternal ethnicity/race (non-Hispanic white, non-Hispanic Black, Hispanic, Other), maternal education (less than high school, high school/GED, some college/associates degree/trade school, bachelor's degree, graduate degree), maternal smoking (yes, no), and child year of birth (1998-2004, 2005-2009, 2010-2014, 2015+)
*Of those excluded: 38% preschool and 62% short form

Table S9: Association Between Maternal Dietary Patterns During Pregnancy and SRS T-scores

	n	Crude, T score (ß, 95% CI)	Adjusted, T score (ß, 95% CI)	Test of trend (p)
EDIP	2433			
Q1	608	0 (reference)	0 (reference)	0.2
Q2	608	-1.00 (N/A, 1.41)	-0.00 (-1.51,0.70)	
Q3	609	-0.00 (-16.40, 6.90)	-0.00 (-1.00,0.80)	
Q4	608	-0.00 (N/A, 0.00)	1.00 (-0.49,1.46)	
AHEIP	1671			
Q1	417	0 (reference)	0 (reference)	0.02
Q2	418	-2.00 (-8.48, 8.48)	0.00 (-0.93,0.92)	
Q3	418	-2.00 (-2.00, 8.04)	*-0.50 (-1.56,0.58)*	
Q4	418	-3.00 (-3.00, N/A)	*-1.00 (-1.65,0.02)*	
HEI	2876			
Q1	719	0 (reference)	0 (reference)	0.11
Q2	719	0.00 (-5.09, 2.09)	-1.00 (-1.78,-0.04)	
Q3	719	0.00 (-5.09, 10.09)	-2.00 (-2.62,-1.04)	
Q4	719	1.00 (-5.09, 2.09)	-1.00 (-1.96,-0.24)	

Modeled using quantile regression fixed at 50th percentile of SRS total T-score
Adjusted: maternal age, maternal pre-pregnancy BMI, child sex (male, female), cohort type (familial/not), maternal ethnicity/race (non-Hispanic White, non-Hispanic Black, Hispanic, Other), maternal education (less than high school, high school/GED, some college/associates degree/trade school, bachelor's degree, graduate degree), maternal smoking (yes, no), and child year of birth (1998-2004, 2005-2009, 2010-2014, 2015+)
Energy Adjusted: maternal age, maternal pre-pregnancy BMI, child sex (male, female), cohort type (familial/not), maternal ethnicity/race (non-Hispanic White, non-Hispanic Black, Hispanic, Other), maternal education (less than high school, high school/GED, some college/associates degree/trade school, bachelor's degree, graduate degree), maternal smoking (yes, no), and child year of birth (1998-2004, 2005-2009, 2010-2014, 2015+)
